# Mango anthracnose disease: the current situation and direction for future research

**DOI:** 10.3389/fmicb.2023.1168203

**Published:** 2023-08-24

**Authors:** Aboagye Kwarteng Dofuor, Naa Kwarley-Aba Quartey, Angelina Fathia Osabutey, Akua Konadu Antwi-Agyakwa, Kwasi Asante, Belinda Obenewa Boateng, Fred Kormla Ablormeti, Hanif Lutuf, Jonathan Osei-Owusu, Joseph Harold Nyarko Osei, William Ekloh, Seyram Kofi Loh, Joseph Okani Honger, Owusu Fordjour Aidoo, Kodwo Dadzie Ninsin

**Affiliations:** ^1^Department of Biological Sciences, School of Natural and Environmental Sciences, University of Environment and Sustainable Development, Somanya, Ghana; ^2^Department of Food Science and Technology, Faculty of Biosciences, College of Science, Kwame Nkrumah University of Science and Technology, Kumasi, Ghana; ^3^Department of Agribusiness, School of Business, Presbyterian University, Abetifi-Kwahu, Ghana; ^4^Entomology Division, Cocoa Research Institute of Ghana, Akim Tafo, Ghana; ^5^Coconut Research Program, Oil Palm Research Institute, Council for Scientific and Industrial Research, Sekondi-Takoradi, Ghana; ^6^Crop Protection Division, Oil Palm Research Institute, Council for Scientific and Industrial Research, Kade, Ghana; ^7^Department of Physical and Mathematical Sciences, School of Natural and Environmental Sciences, University of Environment and Sustainable Development, Somanya, Ghana; ^8^Department of Parasitology, Noguchi Memorial Institute for Medical Research, College of Health Sciences, University of Ghana, Accra, Ghana; ^9^Department of Biochemistry, School of Biological Sciences, College of Agriculture and Natural Sciences, University of Cape Coast, Cape Coast, Ghana; ^10^Department of Built Environment, School of Sustainable Development, University of Environment and Sustainable Development, Somanya, Ghana; ^11^Soil and Irrigation Research Centre, College of Basic and Applied Sciences, School of Agriculture, University of Ghana, Accra, Ghana

**Keywords:** mango disease, anthracnose, epidemiology, detection, host range, management strategies, *Colletotrichum gloeosporioides*

## Abstract

Mango anthracnose disease (MAD) is a destructive disease of mangoes, with estimated yield losses of up to 100% in unmanaged plantations. Several strains that constitute *Colletotrichum* complexes are implicated in MAD worldwide. All mangoes grown for commercial purposes are susceptible, and a resistant cultivar for all strains is not presently available on the market. The infection can widely spread before being detected since the disease is invincible until after a protracted latent period. The detection of multiple strains of the pathogen in Mexico, Brazil, and China has prompted a significant increase in research on the disease. Synthetic pesticide application is the primary management technique used to manage the disease. However, newly observed declines in anthracnose susceptibility to many fungicides highlight the need for more environmentally friendly approaches. Recent progress in understanding the host range, molecular and phenotypic characterization, and susceptibility of the disease in several mango cultivars is discussed in this review. It provides updates on the mode of transmission, infection biology and contemporary management strategies. We suggest an integrated and ecologically sound approach to managing MAD.

## 1. Introduction

Mango anthracnose disease (MAD) is a worldwide disease that is extremely destructive to mangoes before and after harvest. The disease damages the infected mango trees, leading to low yield and quality of fruits. MAD can cause a 100% loss of yield in orchards that are not well taken care of and where the environment is good for the disease to spread. The disease occurs in almost all regions that produce the crop, and its high economic losses have prompted a wealth of research focusing on postharvest losses (Cheng et al., 2022; Silué et al., 2022), damage (Wu et al., 2022), characterization (Ismail and El-Ganainy, 2022), diagnosis and classification (Alberto et al., 2022; Patil et al., 2022; Prabu and Chelliah, 2022), genomics (Ciofini et al., 2022; Kumari et al., 2022), control (Evangelista-Martínez et al., 2022; Liang et al., 2022) and resistance management (Janamatti et al., 2022).

The *Colletotrichum* species complex is responsible for the fungal disease known as MAD. Weir et al. (2012) used morphological and molecular techniques to determine that roughly twenty-two species and one subspecies make up the *C. gloeosporioides* complex. However, several *C. gloeosporioides* isolates in various locations worldwide are characterized by characteristic black, expanding lesions on mango plant parts, including fruits, leaves, flowers, petioles, twigs, and stems. Many of these isolates, including *C. alienum*, *C. fructicola, C. siamense*, *C. tropicale*, and *C. asianum*, have significantly damaged millions of mango trees in Mexico, China, and India (Weir et al., 2012; Li et al., 2019; Tovar-Pedraza et al., 2020). In the United States, *C. aeschynomenes*, *C. musae*, and *C. nupharicola* are associated with anthracnose in mangoes (Su et al., 2011; Weir et al., 2012). Several articles such as Ciofini et al. (2022), Paudel et al. (2022), Jenny et al. (2019), Honger et al. (2015), Nelson (2008), and Arauz (2000) have reviewed MAD. However, all the reviews have a narrow focus on particular geographical areas, including Ghana (Kankam et al., 2022) and India (Maske et al., 2022). Though the economic impacts and management of the disease in many countries have been investigated by different authors (e.g., Arauz, 2000), these studies were confined to a small geographical region.

In contrast to previous articles on mango anthracnose, this review provides a concise summary of the current state of knowledge about MAD from a global viewpoint, including the most up-to-date information on its history, economic importance, epidemiology, early detection methods and management strategies. It highlights the rapid diagnosis of MAD using a computer vision system and a species-specific PCR assay to slow down the spread of the disease into disease-free areas. The biological relationships between the pathogen, environmental conditions, and host plant susceptibility have been better understood due to early detection technologies, genome sequencing, and machine learning.

This review is well-timed because MAD is spreading rapidly to disease-free countries like Indonesia (Benatar et al., 2021), Vietnam (Li et al., 2020) and Cuba (Manzano León et al., 2018). MAD continues to be a severe problem in many regions; for instance, the detection of new strains in Peru (Vilcarromero-Ramos et al., 2022) and China (Ahmad et al., 2021) threatens the livelihood of millions of people that depend on the crop. The potential of different strains of the fungus coexisting within a country; for example, in the northeast of Brazil, where *C. asianum, C. fructicola, C. tropicale, C. karstii,* and *C. dianesei* are present (Lima et al., 2013a,b) highlights the importance of this review. We also provide an update on the taxonomic status of the *Colletotrichum* taxa linked with various MAD, which has changed since the introduction of molecular techniques.

## 2. History and geographical distribution of mango anthracnose

**Figure 1:**
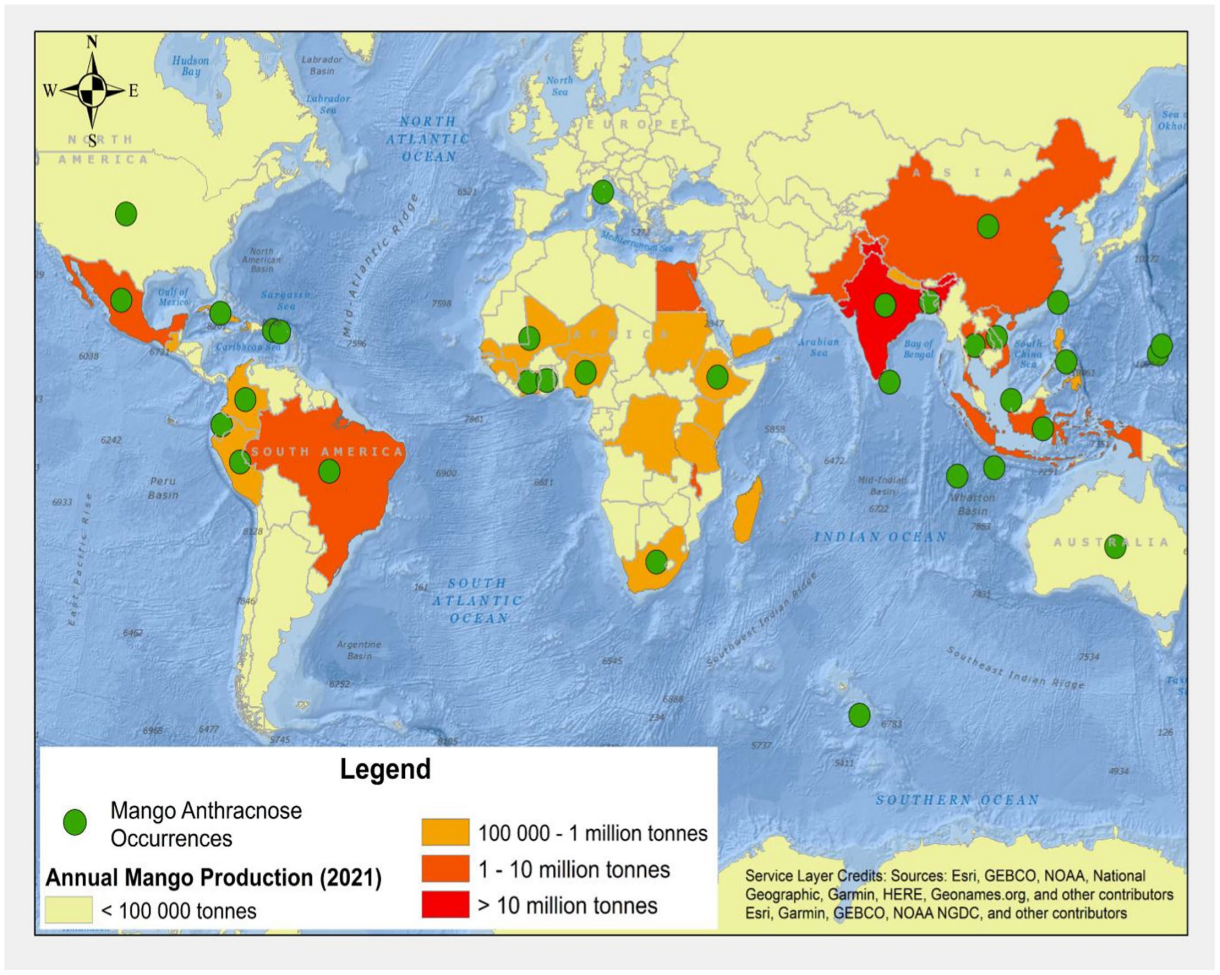
Global map of mangoes production and known distribution records for mango anthracnose [refer to Table 1 for further details (FAOSTAT, 2021)].

Globally, *Colletotrichum* species was first reported by Corda (1831). Since then, the disease has been infecting many crops and trees, including mangoes worldwide (Figure 1; Ciofini et al., 2022). MAD occurs in several countries, including Côte d’Ivoire, Ethiopia, Ghana, Nigeria, South Africa in Africa; Australia in Oceania; Bangladesh, China, India, Indonesia, Taiwan in Asia; and Colombia, Mexico, and Peru in the Americas. The disease mainly affects the leaves, tissues, peduncle, pedicle, twig, stem, fruit, and pulp. Different species of MAD may be part of a complex or just a singleton, and these fungal infections have been reported in different countries. Members of the *Colletoctrichum* genus are the predominant pathogens that cause MAD. They are made up of about 200 species that are tentatively placed into 15 species complexes and singletons (Talhinhas et al., 2018; Guevara-Suarez et al., 2022). Several mango species tolerant to anthracnose diseases have been determined through screening after inoculating these mango species with *Colletotrichum asianum* (Grice et al., 2022). Moreover, many working groups from different geographical regions or countries, such as Ethiopia and Nigeria, while studying the MAD distribution, symptoms, pathogenicity, etiology, incidence, and severity, have also reported MAD infections of different parts of mango (Awa et al., 2012; Chala et al., 2014; Tucho et al., 2014; Benatar et al., 2021). Ismail et al. (2015) reported the recovery of *C. gloeosporioides*, *C. kahawae* subsp. c*iggaro*, and *C. karstii* for the first time in Italy.

Reports of the *C. gloeosporioides* species complex, including *C. alienum, C. asianum, C. fructicola, C. siamense*, and *C. tropicale*, were identified in Mexico in 2019 (Tovar-Pedraza et al., 2020). Among these, *C. alienum* was reported for the first time in Mexico. The *C. alienum* infections have spread into many anthracnose disease endemic areas; for instance, it was reported in China in 2020 (Ahmad et al., 2021). Another type of MAD discovered in 2019 was the *Colletotrichum scovillei* (Qin et al., 2019). Table 1 further explains the history and distribution of MAD.

**Table 1:** History and geographical distribution of the mango anthracnose disease.

Year	Pathogen/Strain (Species Complex)	Pathogen/Strain (Singleton Species)	Host Part Affected	Distribution	Comment(s)	References
2012	*Colletotrichum gloeosporioides*	*Colletotrichum gloeosporioides*	Mango fruit	Nigeria	First report of *Colletotrichum gloeosporioides* in southwest Nigeria	Awa et al. (2012)
2014	*C. gloeosporioides*	*C. gloeosporioides*	Leaves, fruits	Ethiopia		Tucho et al. (2014)
2014	*C. gloeosporioides*	*C. gloeosporioides*	Mango fruit	Ethiopia		Chala et al. (2014)
2015	*C. gloeosporioides*	*C. gloeosporioides*	Panicles, leaves, fruits	Ghana		Honger et al. (2015)
2015	*C. gloeosporioides*	*C. gloeosporioides*	Mango fruit	Nigeria		Onyeani and Amusa (2015)
2015	*C. gloeosporioides*	*C. asianum*	Mango fruit	South Africa		Sharma et al. (2013)
2015	*C. gloeosporioides C. acutatum*	*C. gloeosporioides C. kahawae* subsp. *ciggaro C. karstii*	Mango leaves and fruits	Italy	First report of *C. gloeosporioides*, *C. kahawaw* subsp. *ciggaro, C. karstii* in Italy	Ismail et al. (2015)
2018	*C. gloeosporioides*	*C. gloeosporioides*	Panicles, leaves, branch terminals of mango	Bangladesh		Uddin et al. (2018)
2018	*C. gloeosporioides*	*C. asianum, C. fructicola,* and *C. siamense*	Mango leaves	China	First report of these pathogens in the Guangxi province, China	Mo et al. (2018) and Tovar-Pedraza et al. (2020)
2018	*C. gloeosporioides*	*C. gloeosporioides*	Mango flower, mango leaves, mango fruit	India		
2019	*C. gloeosporioides*	*C. gloeosporioides*	Leaves, fruits	Côte d’Ivoire	First reported in 1951 and identified in 1979	Dembele et al. (2019)
2019	*C. gloeosporioides*	*C. alienum*	Mango peels, mango fruit pulps	China	First report of this pathogen in China	Ahmad et al. (2021)
2019	*C. gloeosporioides*, *C. boninense*	*C. asianum, C. cliviicola, C. cordylinicola, C. endophytica, C. fructicola, C. gigasporum, C. gloeosporioides, C. karsftii, C. liaoningense, C. musae, C. scovillei, C. siamense* and *C. tropicale*	Mango leaves, mango fruits	China	Aside *C. asianum, C. fructicola, C. scovillei* and *C. siamense*, all the other *C.* spp. were being reported to infect mangoes for the first time in China. However, this was the first report of *C. cordylinicola, C. endophytica, C. gigasporum, C. liaoningense and C. musae* infecting mangoes worldwide	Li et al. (2019)
2019		*C. scovillei*	Mango leaves	China	First report of *C. scovillei* infecting mangoes in China	Qin et al. (2019)
2019	*C. gloeosporioides*	*C. gloeosporioides*	Mango fruit	China		Feng et al. (2019)
2019	*C. gloeosporioides*	*C. gloeosporioides*	Mango fruit	Mexico	First report of antagonistic mechanisms of the marine bacterium, *Stenotrophomonas rhizophila*, against anthracnose disease in mango	Reyes-Perez et al. (2019)
2020	*C. gloeosporioides*	*C., C. asianum, C. fructicola, C. siamense,* and *C. tropicale*	Mango tissues	Mexico	First report of all five species in Mexico and the first report of *C. alienum* MAD worldwide	Tovar-Pedraza et al. (2020)
2021	*C. gloeosporioides*	*C. asianum*	Mango fruit	Indonesia	First report of *C. asianum*	Benatar et al. (2021)
2022	*C. gloeosporioides*	*C. asianum*	Mango leaves	Australia		Grice et al. (2022)
2022	*C. acutatum*, *C. boninense*, *C. gigasporum*, *C. gloeosporioides*, *C. orbiculare*	*C. acutatum*, *C. godetiae*, *C. laticiphilum*, *C. tamarilloi*	Mango flower, mango leaves, mango fruit	Colombia, Equador (Equador – *Colletotrichum acutatum*, *C. tamarilloi*)		Guevara-Suarez et al. (2022)
2022	*C. gloeosporioides, C. acutatum*	*C. gloeosporioides,* and *C. acutatum*	Mango flower, mango leaves, mango fruit, twig	India		Kadam et al. (2002)
2022	*C. gloeosporioides*	*C. asianum*	Mango fruit	Peru	First report of *C. asianum* infecting mangoes in Peru	Vilcarromero-Ramos et al. (2022)
2022	*C. gloeosporioides*	*C. gloeosporioides*	Mango fruit	Taiwan		Liang et al. (2022)

## 3. Economic importance of mango anthracnose

MAD represents the most severe fungal disease restricting the cultivation and commercialization of mango fruits internationally. *C. gloeosporioides*, the disease’s causative organism, represents one of MAD’s most economically important agents that can impact the sorting, packaging, shipping, storage, and sale of mango fruits (Bhagwat et al., 2015). In many regions, pre-harvest infections from organisms like fungi, viruses, bacteria, and nematodes have led to low mango harvests (Chowdhury and Rahim, 2009). MAD is one example of a postharvest disease that drastically reduces fruit quality, leading to substantial economic losses (Akem, 2006). The economic and scientific importance of postharvest damage from MAD is that it lowers fruit quality and shelf life, thereby influencing export quality standards (Kankam et al., 2022).

MAD typically causes yield losses through a decline in either the quantity or quality of harvested mango fruits. According to local reports, MAD poses a severe threat to Ghana’s mango crop, causing a 30% output loss in one of its districts (Honger et al., 2014). Similar to how anthracnose disease is responsible for roughly 39% of yield loss in mango cultivation in India (Prakash and Srivastava, 1987). MAD causes a 30–60% annual loss of mango, with potential damage of 100% under ideal conditions (Kamle and Kumar, 2016). In Gondunglegi, Indonesia, yield loss due to anthracnose was reported to be 50.28% by Kumari et al. (2017) and Arauz (2000), while in Himachathe l Pradesh, India, the postharvest loss was reported to be 29.6% from 1990 to 1992 (Sharma and Verma, 2007). In Hyderabad, 20 to 30% of mango fruits rotted due to *C. gloeosporioides*. According to Hossain and Ahmed (1994), MAD and stem end rot diseases account for a 25–30% loss in mango yield in Bangladesh. Mangoes in Thailand were lost at a rate of 62.8% during harvest and 63.2% in the markets due to MAD, according to research by Sardsud et al. (2003). Anthracnose can cause a 30–60% annual loss in China’s mango harvest (Li et al., 2019).

Severe problems in nurseries and orchards may appear under crowded and moist conditions (Lai and Simon, 2013). The incidence may increase significantly under favorable environmental conditions. Damage to foliage, a reduction in flower production, and yield losses have all been attributed to *C. gloeosporioides.*

Humid climates are also ideal for the spread of anthracnose fungus (Arauz, 2000; Akem, 2006). The disease has a prevalence of about 100% in fruits grown in damp or very humid environments (Arauz, 2000; Akem, 2006; Chowdhury and Rahim, 2009). MAD can also induce postharvest deterioration, leading to the fruit being rejected by consumers.

MAD is widely recognized as a significant challenge to southern Ethiopia’s mango farming business’s sustainability (Chala et al., 2014). Mango exporting countries like Ghana suffer the most as a result of the disparity in revenue between export and local markets, despite this, producers and sellers continue to sell low-quality fruits on the local market (Kankam et al., 2022).

The expense of controlling MAD could have repercussions for the economy. Farmers have used a variety of fungicides to prevent the fungus from spreading. Smallholder farmers, who may lack the capital to invest in such suggested management tactics, may profoundly feel the economic effects of these controls. In addition, fungi can become resistant to fungicides if too many are used, necessitating the use of more potent or hazardous compounds.

## 4. Symptoms of mango anthracnose

**Figure 2:**
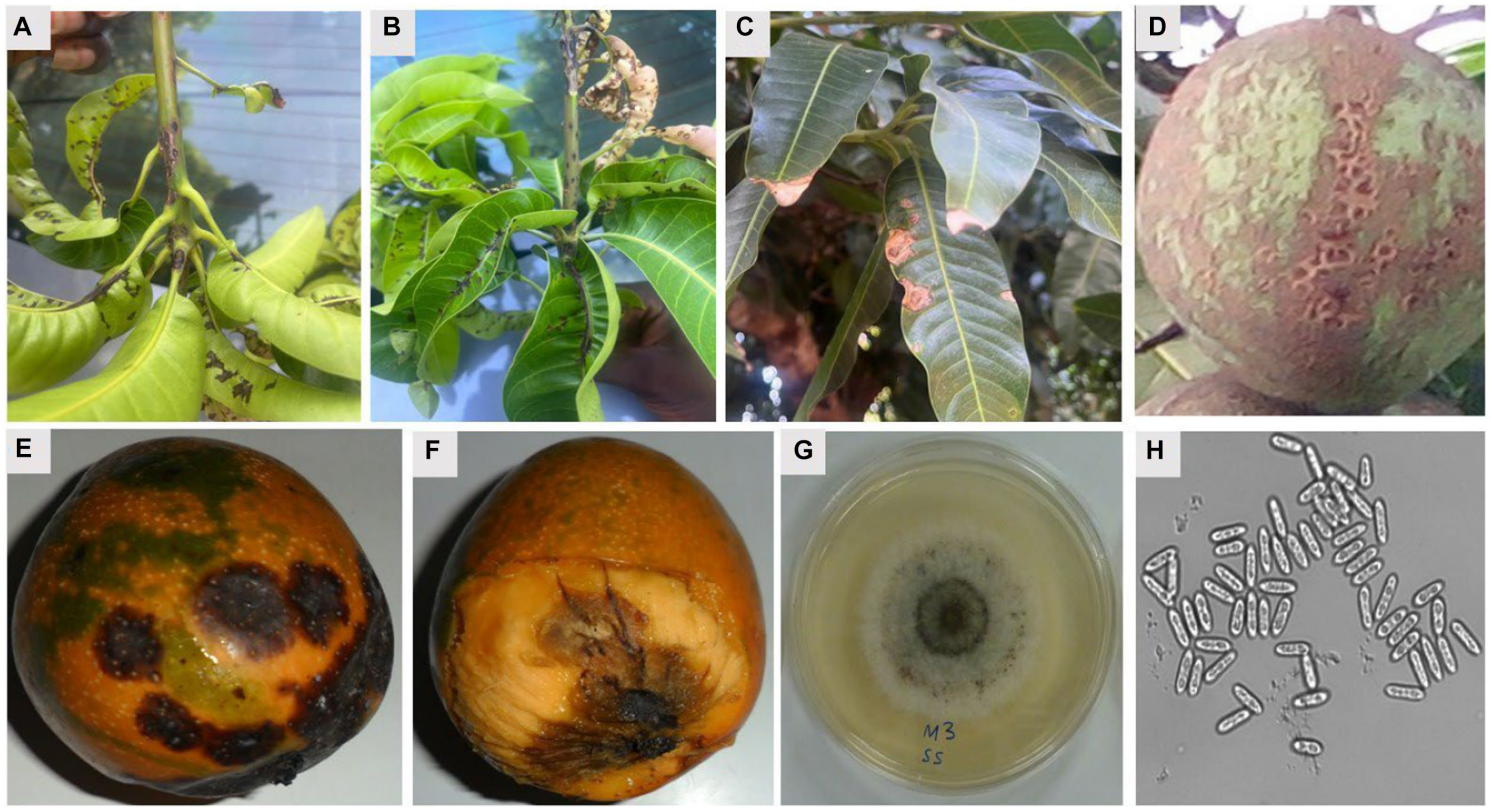
Symptoms of mango anthracnose disease and cultural and morphological features of *C. gloeosporioides* causing anthracnose disease of mango. **(A,B)** Symptomatic leaves and twigs, **(C)** symptomatic mature leaves, **(D)** the alligator skin effect symptoms on unripe fruit, **(E)** typical sunken spot symptoms of mango anthracnose, **(F)** a ripe fruit with the pericarp removed to show the penetration of the dark lesions of the anthracnose disease on the fruit pulp, **(G)** white mycelial growth on PDA showing presence of acervuli ring in the middle of the culture, and **(H)** short conical spores with rounded edges **(H)**. Mg × 200.

MAD is among the most widespread diseases that can attack mango at any time, whether when it is still in the field, in transportation, or storage. Mango orchards may show symptoms of the disease on the mango trees’ leaves, twigs, and fruits (Figure 2). It can also be found on the blossoms (Uddin et al., 2018). The disease’s symptoms show up as oval or irregular brown to deep brown sunken point-sized spots of varying sizes spread all over the leaf surface, most prominently on young leaves but also on older leaves. As the leaves age, these spots develop into larger lesions that are either circular or irregular in shape and surrounded by a red halo (Qin et al., 2019).

The fungus can produce blossom blight on the inflorescence. Lengthy lesions ranging from dark grey to black can appear in the stalk. The panicles and open flowers also develop tiny black spots that eventually grow and kill the plant. Flowers that have been blighted become brittle and dark brown to black (Alemu et al., 2014). The fungus sometimes invades the twigs, stems and branches of the mango tree. Symptoms on twigs manifest as small, expanded oval and necrotic lesions that eventually consolidate and disperse. The fungus can invade twigs during severe infections and cause dieback (Tovar-Pedraza et al., 2020).

MAD is most common on immature fruits and during transport and storage but can occur at any stage during the fruit’s life cycle. The young fruits are either aborted or mummified, and infection on larger fruits may remain latent or dormant until the ripening of the fruit, where black, sunken necrotic lesions appear on the fruit peel and increase rapidly in size (De Souza et al., 2013). Tear staining, caused by spore-laden water droplets from diseased twigs and leaves spreading across the fruit and infecting the surface, is another symptom of anthracnose damage to fruit (Mabbett, 2014). Several other diseases attack the mango plant aside from anthracnose. Some of these diseases have symptoms similar to anthracnose, and others have distinct symptoms. A couple of such equally important diseases of mango are compared with anthracnose.

Alternaria leaf spot disease is similar to anthracnose disease because it mostly attacks the young, tender leaves and is characterized by brown spots. Anthracnose, however, shows up as irregular, oval-shaped spots all over the leaf’s surface while in Alternaria leaf spot, typical round spots are uniformly distributed across the leaf’s lamina and more noticeable on the leaf’s underside. Symptoms of bacterial canker disease on leaves include yellowing and eventual leaf drop, as well as wet, irregular to angular, elevated lesions that are sometimes clustered near the leaf apex. While anthracnose leaf spots are large, scab disease spots are tiny and cause the leaf to become distorted and wrinkled before falling off prematurely. A powdery fungus growth coating on the leaves also characterizes powdery mildew disease.

Black-banded or black velvety mycelial growth on leaf veins and midribs is a telltale sign of disease (Gautam et al., 2017), while red rust is characterized by small, circular lesions that appear on the top leaf surfaces and coalesce toward the midribs (Patrice et al., 2020). Stem end rot is a disease that affects fruits and causes these fruits to turn black at the stem end, eventually spreading to cover about half of the fruit. Though the damaged skin retains its firmness, the rot sets into the pulp, which gives off a foul odor. Additionally, water-soaked sores on fruits progressively develop into cankers, which break open and release a gummy slime due to bacterial canker disease. Conversely, MAD causes black spots, which are initially spherical, then transform into big irregular blotches across the entire fruit while in storage. Large, gaping crevices form at these sites, allowing the fungus to eat its way deep into the fruit (Sefu et al., 2015).

The necrotic regions on the twigs become increasingly longer and black due to the twig blight disease. The leaves begin to drop and eventually dry out and fall off. Very immature branches begin to dry out from the tips down. Dieback disease causes twigs to wilt and die from the top down, especially in older trees, and then leaves to wilt and die, giving the impression of a fire scorch (Saeed et al., 2017). Young, green twigs get discolored and black, eventually dying from the top down (Malik et al., 2014). Mango twigs eventually succumb to MAD during extremely severe outbreaks and vice versa.

For powdery mildew, the distinguishing symptoms of the disease are whitish powdery fungus growth coating on flowers. Affected flowers and young fruits that have reached marble size may drop prematurely (Ajitomi et al., 2020). The panicles and open flowers also develop tiny black spots that eventually grow and kill the plant. Flowers affected by anthracnose are dry and dark brown to black in hue (Rosman et al., 2019). It is estimated that the disease causes 30–60% damage economically, which can climb to 100% in fruits produced during humid seasons or at the commencement of rains (Kamle and Kumar, 2016).

MAD causes the most harm in wet environments during the flowering and fruit-setting periods. Flowering and early fruit development are susceptible to mango infections (Sarkar, 2016). Some cutting-edge strategies for early diagnosis of anthracnose disease in mangoes include the application of Modified Rotational Kernel Transform Features (Ullagaddi and Raju, 2017), computational biology, and image processing analytic methods (Khan et al., 2019).

## 5. Biochemical and molecular characterization of pathogen

Conserved signal transduction pathways control fungi growth, development, and reproduction (Cannon et al., 2012; Gan et al., 2013). The mitogen-activated protein (MAP) kinase cascades have been shown to be responsible for the infection-related morphogenesis in pathogenic fungi like *C. gloeosporioides* (Yong et al., 2013; Zhou Z. et al., 2017). Yong et al. (2013), in their study isolated and characterized the MAK kinase gene *Cgl-SLT2* from *C. gloeosporioides*, and indicated the full involvement of MAPK in conidiation, appressorium formation, polarized growth, and the pathogenicity of the filamentous fungus. The MAPK cascade, which consists of three conserved kinases, the MAP kinase (MAPK), MAP kinase kinase (MEK), and MAP kinase kinase kinase (MEKK), is a pivotal signaling pathway sensing and relaying extracellular signals to control gene expression (Yong et al., 2013). The fungal MAPK cascade together with the calcium-calcineurin pathway, which functions via calmodulin, the Ca^2+^ binding protein, calcineurin, and the calmodulin-dependent serotonin-threonine phosphatase (Zhou Z. et al., 2017), targets a wide range of downstream effectors including enzymes, other proteins, and transcription effectors. Thus, triggered by a host of stimuli, the two pathways regulate basic cell function and stress response. Like other pathogenic fungi that attack plants, *C. gloeosporioides* secret proteolytic enzymes such as pectate lyase (PEL), endopolygalacturonase (PG) and pectin lyase (PNL) (Zhou Z. et al., 2017). These essential pathogenic functionaries can depolymerize polysaccharides present in the primary cell wall of the host during infection and colonization (Zhou Z. et al., 2017). Zhou Z. et al. (2017) reported that *C. gloeosporioides* required the ABC (ATP-binding cassette) protein CgABCF2 for appressorial formation and plant infection as well as for sexual and asexual reproduction.

Many of the studies conducted on *C. gloeosporioides* depend on evidence from previous works which stated that the sequence data in the domain 2 (D2) region of the rDNA could efficiently reveal information about the type of leucyl-tRNA synthetase (LARS) and relationships within *C. gloeosporioides* (Cannon et al., 2012; Sharma et al., 2013). A recent study caused mutations within the WT strain W16 of *C. gloeosporioides* via ATMT (*Agrobacterium tumefaciens*-Mediated Transformation) to study genes associated with conidiation (Wu et al., 2016). The insertional mutagenesis populations were then isolated, and conidiation assays were conducted on 59 conidial production-variation transformants (Wu et al., 2016). The study conclusively found the oligopeptide transporter (Opt) to be involved in *C. gloeosporioides* conidiation. The team also identified 19 putative genes including *CgOPT2* (oligopeptide transporter protein), *CgMCT1* (monocarboxylate transporter protein), *CgCOP9* (Cop9 signalosome subunit 6 protein), *CgMRP1* (multidrug resistance- associated protein 5), *CgRXT2* (Rxt2-like protein) and *CgRRN3* (specific transcription initiation factor RRN3). Interestingly, while the WT strain W16 contained 19 genes, 11 of these genes were not detected in T-DNA insertional mutants that were also sequenced (Wu et al., 2016). The team inferred from the observational differences in phenotypes that variations in conidiation and production of albino hyphae could be attributed to the absence of those 11 genes (Wu et al., 2016).

Early in the 20th century, research teams successfully isolated and characterized phenotypes of *C. gloeosporioides* using morphological features. Relying on morphology of appressoria and conidiomata conidia dimensions, three biological groups within the *C. gloeosporioides* complex were determined (Sharma and Kulshrestha, 2015). These were *C. gloeosporioides* var. *gloeosporioides* which had unlobed or slightly lobed appressoria and conidial widths of 4.5–5.5 μm; *C. gloeosporioides* var. minor of similar appearance but with mean conidial widths of 3.0–4.2 μm, and a third phenotype that closely matches the definitions classifying *C. crassipes* with lobed appressoria and conidial widths of 4.5–5.5 μm (Wu et al., 2016).

On the molecular level, phylogenetic relationships in the *Colletotrichum* spp. have been successfully identified through sequencing in the internal transcribed spacers 1 and 2 (*ITS1* and *ITS2*) regions and glyceraldehyde-3-phosphate dehydrogrenase (GAPDH) gene (Sharma and Kulshrestha, 2015; Pardo-De la Hoz et al., 2016). Using an additional genomic region, *ApMat,* the three strains identified in the *C. gloeosporioides* complex were refined and confirmed (Doyle et al., 2013; Pardo-De la Hoz et al., 2016). From work in the intergenic regions of *apn2* and *MAT1-2-1* (ApMat) genes, 22 phenotypes (Supplementary Table S1) have been identified (Cannon et al., 2012), and four distinct phylogenies (*C. fructicola*, *C. frgariae sensu stricto*, *C. melanocaulon* and *C. jasmine-sambac*) within the complex described (Doyle et al., 2013). Subsequently, another team using the same approach confirmed that these four and five potentially novel *Colletotrichum* lineages, yet to be assigned specie names, were associated with anthracnose in mango tissues (Sharma et al., 2013).

Intervention strategies against *C. gloeosporioides* have been explored as molecular knowledge of the pathogenic fungus delves deeper. For instance, Yong et al. (2013) showed conclusively that the MAPK gene *Cgl-SLT2* is required for appressorium formation, conidiation and pathogenicity in *C. gloeosporioides*. Subsequently, the mutant *Cgl-slt2* showed defective hyphae formation and sporulation compared to the wild type. Other mutagenic studies are exploring using T-DNA (transferred-DNA) to yield fewer pathogenic phenotypes.

## 6. Mode of transmission and infection of pathogen

Pathogenic fungi of plants need to align with the seasonal growth stages of the host plant for a successful transmission and infection cycle. For instance, since most plants shed leaves and remain dormant during the autumn and winter seasons, plants’ pathogenic fungi typically adopt corresponding dormancy strategies until spring (Marcianò et al., 2021). These strategies include the production of spores, the development of sclerotia, hiding within the host plant, finding shelter in the ground, or moving to another active host plant during adverse conditions (Marcianò et al., 2021). When conditions become favorable, spores may be transmitted by water, wind, or animals to a susceptible host for infection.

At least three major infection strategies are implicated in the molecular pathogenesis of plant fungal pathogens. Fungal strategies may involve preventing host recognition, inhibiting host defense mechanisms, and hijacking host cellular machinery (Rodriguez-Moreno et al., 2018). Specifically, to penetrate the plant host, pathogenic fungi may secrete cell wall-degrading carbohydrate-active enzymes such as glycoside hydrolases, glycosyltransferases, polysaccharide lyases, carbohydrate esterases, and redox enzymes (Lombard et al., 2014). Plant fungi may also modify the components of their cell walls through the accumulation of specialized carbohydrates to prevent degradation by plant chitinases (Fujikawa et al., 2012) or the secretion of carbohydrate-binding effectors to inhibit chitin-mediated host response (de Jonge et al., 2010; Sanchez-Vallet et al., 2013).

Moreover, pathogenic fungi can hijack the host defense system by subversion of reactive oxygen species, modification of host pH, regulation of hormone signaling, and inhibition of host proteases (Rodriguez-Moreno et al., 2018). In most of these infection cycles, fungal effector proteins, secondary metabolites, host-specific toxins, and small RNAs (sRNA) are increasingly gaining prominence as critical regulators and mediators in the mechanism of molecular pathogenesis (Rodriguez-Moreno et al., 2018).

**Figure 3:**
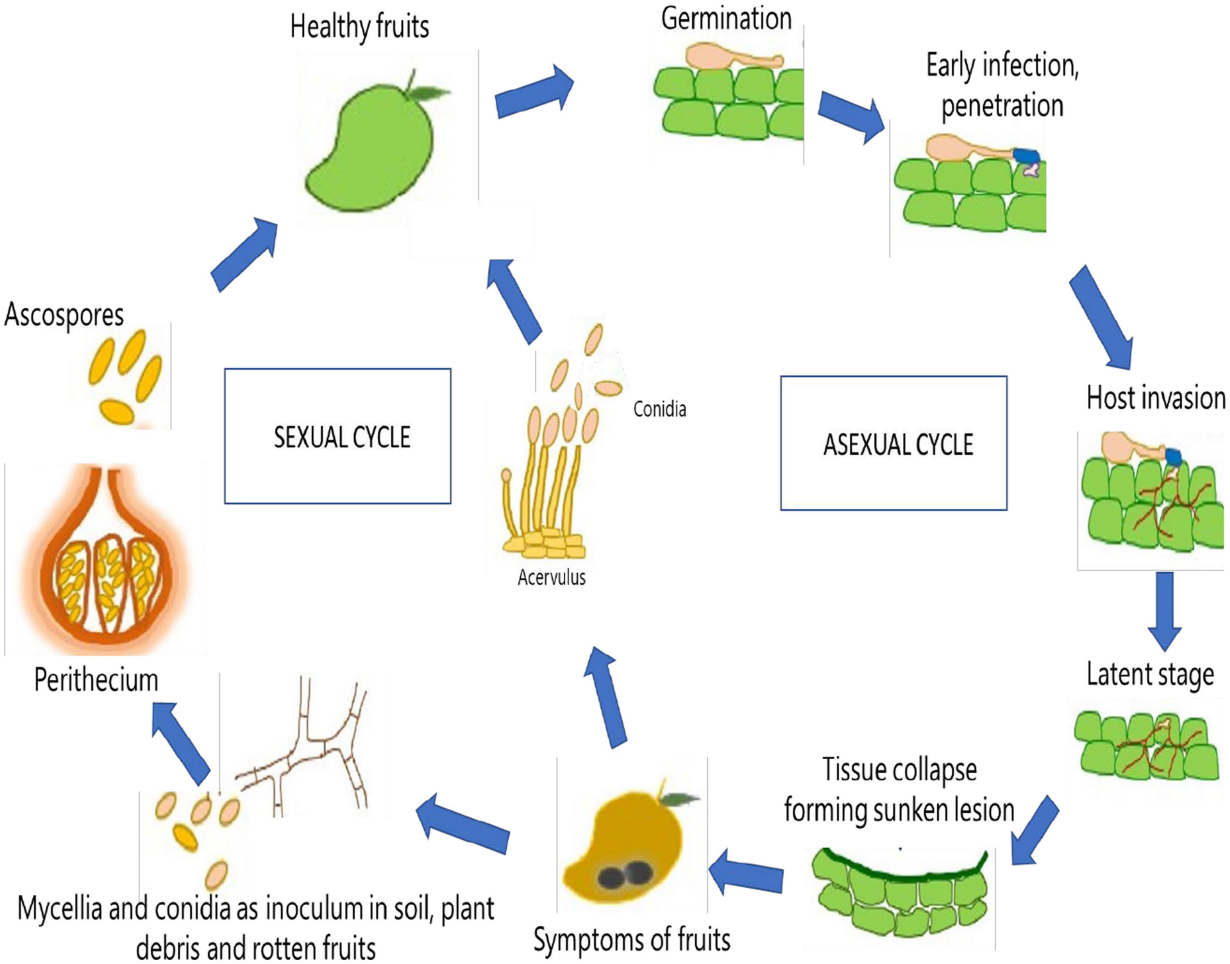
Life cycle of *Colletotrichum gloeosporioides* in Mango (*Mangifera indica*).

*Colletotrichum gloeosporioides* is typically dormant in the dry season, especially during extremes of temperature, low humidity, and sunlight (Sharma and Kulshrestha, 2015). However, optimal growth of the pathogen is favored at high humidity, pH range of 5.8 to 6.5, and temperature of 25–28°C (Sharma and Kulshrestha, 2015). These favorable conditions stimulate the release of spores from acervuli of seeds, leaves, fruits, or trash of hosts. Dispersal of spores may occur through air currents, insects, water splash or other forms of direct transmission (Sharma and Kulshrestha, 2015). After successful dispersal, *C. gloeosporioides* utilizes a hemibiotrophic mode of infection to penetrate, develop and spread within a susceptible host plant (Münch et al., 2008). Through this mode, the infection of host plants usually consists of two stages: penetration and colonization (Marcianò et al., 2021). *Melanized appressoria* are initially formed to aid in host penetration and harmless formation of primary hyphae in a biotrophic phase of infection, followed by the secondary formation of hyphae in a necrotrophic phase that is potentially deleterious to the host (Münch et al., 2008). This leads to the spread of spores on the surface of infected tissues that are subsequently dispersed to repeat the transmission and infection cycle of the pathogen (Figure 2). Thus, the disease moves through a cycle of dissemination of asexual spores (conidia), inoculation of spores into susceptible part of the host, development of symptoms in fruiting bodies (acervuli), infection of a host, further development of disease, reproduction of pathogen, and survival of pathogen (Paudel et al., 2022). *Glomerella cingulata*, the name typically given to the sexual stage (teleomorph) of the same pathogen, may induce dark, long-necked perithecia with clavate asci that are relatively rarely observed (Figure 3).

Several *C. gloeosporioides* genes play critical roles in host defense mechanisms during infection. Most of these genes interact with a pH-responsive transcription factor (pacC) that regulates the expression of approximately 5% fungal genome involved in transport, oxidative damage, and cell wall degradation (Alkan et al., 2013). The expression of pectate lyase by *pelB* induces the degradation of the plant cell wall in a pH-dependent manner under the regulation of pacC (Drori et al., 2003; Kramer-Haimovich et al., 2006). A *PepCYP* product homologous to cytochrome P450 with a heme-containing domain involved in host defense mechanisms during pathogen invasion and colonization is also expressed in a ripening–dependent manner by *C. gloeosporioides* (Oh et al., 1999).

Several genes also mediate pathogenesis in the fungus. CgRac1 protein regulates morphogenesis, nuclear division, and germination via potential concentration in conidia and hyphal tips (Nesher et al., 2011). The expression of *cgOPT1* varies in resting and germinating spores during mycelia development (Chagué et al., 2009). Several nitrogen-metabolism-related genes (*GDH2, GS1, GLT,* and *MEP*) are differentially expressed to aid in the induction of ammonia accumulation and fungal pathogenicity (Miyara et al., 2012). The expression of Bcl-2 protein plays a role in cell death and survival of the fungus through processes such as pathogenicity, conidial germination, and mycelium growth (Barhoom and Sharon, 2007). CgCTR2, a putative vacuolar copper transporter, is critical in cellular copper balance during the initial stages of pathogenesis (Barhoom et al., 2008). The expression of pel1 and pel2 proteins also plays vital roles in pathogenesis during the neurotropic phase of fungal infection (Shih et al., 2000). The expression of the *cgDN3* gene of *C. gloeosporioides* is stimulated during the early stages of infection to aid in the modulation of a hypersensitive-like response by a compatible host (Stephenson et al., 2000). The *chip6* gene plays an essential role in conidial germination and appressorium formation (Kim et al., 2002). Moreover, two pectin lyase genes are differentially expressed during fungal pathogenesis: *pnl-2* is highly expressed in the necrotrophic phase of infection, and *pnl-1* may be observed in both necrotrophic and biotrophic phases (Wei et al., 2002).

## 7. Host range

The pathogen responsible for severe anthracnose infections in Brazil’s southeast was identified, and disease susceptibility among a global collection of mango germplasm was assessed, based on a survey. *Colletotrichum* was the most common pathogen of commercial mangoes in the region, and the cultivars ‘Ubà,’ ‘Quinzenga,’ ‘Amarelinha da Sementeira,’ ‘Aroeira,’ and ‘Correjo’ were particularly vulnerable to *C. asianum* infections (Vitale et al., 2020). The disease is notably less severe on ‘Ourinho’ and ‘Lita’ cultivars but less on ‘Mallika’. None of the accessions tested was resistant, and commercial cultivars generally cannot deliver appropriate qualitative and quantitative yields under humid environmental conditions without regularly applying fungicide sprays (Arauz, 2000). Mango (‘Palmer,’ ‘Keith,’ and ‘Tommy Arkins’) from the orchards of Northeastern Brazil were used to test the virulence of five species of *Colletotrichum* (*C. asianum, C. dianesei, C. fructicola, C. karstii,* and *C. tropicale*). Neither the ‘Keith’ nor the ‘Palmer’ cultivars were affected by *C. karstii*, suggesting that it is not pathogenic to these species (Lima et al., 2015). The Tommy Atkins cultivar was more tolerant to infection from all *Colletotrichum* species tested, and the alternate host plants were all susceptible to the *Colletotrichum* spp. In Australia, ‘Carrie’, ‘Tommy Atkins’, ‘Saigon’ cultivars are resistant to *C. gloeosporioides*, while ‘Kensington Pride’ shows moderate resistance to the disease (Nelson, 2008; Kankam et al., 2022). In the Philippines, ‘Palmer’, ‘Siam’, ‘Velei-Colomban’, and ‘Joe Welch’ are resistant cultivars, whereas ‘Fernandin’, ‘Arumanis’, ‘Edward’, ‘Gedong’, and Tjenkir’ are fairly resistant to the disease. Moreover, ‘Paris’, ‘Fairchild’, and ‘Rapoza’ show resistance to *C. gloeosporioides* in Hawai‘I, with only ‘Haden’ showing moderate resistance to the pathogen. In Florida, the ‘Zill’ shows resistance to *C. gloeosporioides*.

In addition to mango, other hosts of *C. gloeosporioides* include Musa species, avocado (*Persea americana* Mill.) and guava (*Psidium guajava* L.) (Nelson, 2008; Moraes et al., 2013). The pathogen has also been found in apples (*Malus domestica* Borkh.) (Munir et al., 2016), papaya (*Carica papaya* L.) (Maharaj and Rampersad, 2012) and leaves, tubers and seeds of yam (*Dioscorea alata* L.) (Abang et al., 2002). Besides, capsicum, coffee, eggplant, and tomato are susceptible to MAD. Lima et al. (2015) suggest alternative host plants (papaya, banana, guava and bell pepper) are susceptible to *C. asianum*, *C. dianesei*, *C. fructicola*, *C. karstii* and *C. tropicale*. There are many strains of the pathogen that can infect non-mango host plants, which include ornamental lupine (*Lupinus hartwegii* L.), marsh lupine (*Lupinus polyphyllus* Lindl.), various herbs such as angelica (*Archangelica officinalis* Hoffm.), thyme (*Thymus vulgaris* L.) caraway (*Carum carvi* L.) and elder (*Sambucus nigra* L.) (Paulitz, 1995). Anthracnose symptoms have been observed in mango cv. R2E2 when *C. alienum* B.S. Weir and P.R. Johnst., C. kahawae subsp. cigarro B.S. Weir and P.R. Johnst., and *C. theobromicola* Delacr., were isolated from avocados (Grice et al., 2022). In Venezuela, Coffee Berry Borer -infested and -uninfested branches and twigs, as well as ripe and green berries with indications of anthracnose, were all found to be positive for *C. siamense* and *C. alienum* (Castillo et al., 2022). The dragon fruit, or *Hylocereus undatus*, is a species of the *Hylocereus* that often has a white pulp and scarlet or pink skin. One of the most widespread worldwide phytopathogens responsible for postharvest anthracnose in dragon fruits is *Colletotrichum* spp. (Bordoh et al., 2020). The disease infects fruits in the field, during transportation, and during cold storage, thereby reducing the shelf life of the fruits. *Colletotrichum scovillei* causes anthracnose symptoms in bananas, mangoes, wampi (*Clausena lansium*, Rutaceae), and onions (Zhou Y. et al., 2017; Qin et al., 2019; Lin et al., 2020; Lopes et al., 2021).

Though *C. tamarilloi* is associated with tamarillo in Colombia and Ecuador, the fungus also attacks mango and onion in Columbia and Brazil, respectively (Damm et al., 2012; Pardo-De la Hoz et al., 2016; Caicedo et al., 2017; Lopes et al., 2021). In China, *C. gigasporum* was isolated from mango (Li et al., 2019), and the same species attacks coffee in Mexico and China (Cristóbal-Martínez et al., 2017; Cao et al., 2019). In Brazil, *C. gigasporum*, was recorded as a secondary fungal disease on *Annona* spp. (Costa et al., 2019). *Colletotrichum alienum* attacks many crops, including *Aquilaria sinensis* and *Camellia sinensis in China* (Liu et al., 2017, 2020; Ahmad et al., 2021). In Australia, the pathogen has been isolated from Fragaria × ananassa, *Grevillea* sp., and *Nerium oleander* (Liu et al., 2013; Schena et al., 2014; Shivas et al., 2016). Moreover, in New Zealand, the pathogen has been reported from *Malus domestica* (Weir et al., 2012), *Persea americana* in Australia, New Zealand and Israel (Weir et al., 2012; Sharma et al., 2017). *Colletotrichum cigarro* occurs in mango in Colombia and Italy (Ismail et al., 2015; Pardo-De la Hoz et al., 2016). *Colletotrichum asianum* is associated with mangoes but has been reported on avocados (*Persea americana*) in Indonesia (Zhafarina et al., 2021). *Colletotrichum cigarro* has been isolated from mango in Colombia and Italy (Ismail et al., 2015; Pardo-De la Hoz et al., 2016). The species also occurs in plants, such *as Kunzea ericoides* in New Zealand (Weir et al., 2012), *Areca catechu* in China (Zhang et al., 2020) and apple (*Malus domestica*) in Belgium and the USA (Grammen et al., 2019; McCulloch et al., 2020). *C. endophyticum* is associated with mango fruits and leaves but also occurs in *Camellia sinensis* (Wang et al., 2016), and coffee (*Coffea arabica* and *C. robusta*) leaves and fruits (Cao et al., 2019). The pathogen is of grave concern in Southeast Asia to tea, coffee and mango plantations (Talhinhas and Baroncelli, 2021). *C. perseae* is associated with MAD (Hofer et al., 2021) and avocado (Talhinhas and Baroncelli, 2021). *C. queenslandicum* affects papaya and avocado in Australia, cashew in Brazil (Veloso et al., 2018), and coffee in Fiji (Weir et al., 2012). The pathogen has been found in a variety of other host plants, including *Citrus latifolia* in the United States (Kunta et al., 2018), *Licania tomentosa* in Brazil (Lisboa et al., 2018), *Litchi chinensis* in Australia (Anderson et al., 2013; Shivas et al., 2016), *Nephelium lappaceum* in Puerto Rico (Serrato-Diaz et al., 2014; Shivas et al., 2016). *C. liaoningense* attacks mango in China (Li et al., 2019). However, the pathogen can also occur in *Solanum pseudocapsicum* (Liu et al., 2021). In mangoes, larger lesions are observed on hosts at temperatures ranging between 25 and 30°C, though many species exhibit varying thermal requirements for maximal pathogenicity in the fruits (Lima et al., 2015). Table 2 lists a range of mango cultivars with varying levels of resistance and susceptibility.

**Table 2:** Mango cultivars susceptibility to the anthracnose disease.

Country	Resistant cultivar	Resistant level	Susceptible cultivars	Reference
USA	Zill Rapoza Haden Paris Fairchild	Resistant Resistant Moderately resistant Resistant	-	Nelson (2008), Ntsoane et al. (2019), Vitale et al. (2020), and Kankam et al. (2022)
Philippines	Velei-Colomban Fernandin Joe Welch Arumanis Palmer Siam	Resistant Moderately resistant Resistant Moderately resistant Resistant Resistant	Cherakuruasa Kensington Julie Ah Ping Otts Hingurakgoda Peter Passand Carrie	Nelson (2008), Ntsoane et al. (2019), Vitale et al. (2020), and Kankam et al. (2022)
Australia	Caraboa Florigon Carrie Tommy Atkins Kensington Pride Saigon	Resistant Resistant Resistant Moderately resistant Resistant	Willard Neelum Manaranijan	Nelson (2008), Ntsoane et al. (2019), Vitale et al. (2020), and Kankam et al. (2022)
Brazil	Ourinho	Moderately resistant	–	Nelson (2008), Ntsoane et al. (2019), Vitale et al. (2020), and Kankam et al. (2022)
	Lita	Moderately resistant		

## 8. Detection of mango anthracnose

Traditionally, MAD is identified using certain morphological features such as mycelial growth, conidia size, colony color, texture, and presence and absence of setae (Abera et al., 2016; Ashraful et al., 2017). Although these features are still used with other techniques to identify and characterize the causative organisms of the disease, the differences in these physical features are not adequate to separate the different species of *Colletotrichum*. This was confirmed in a study where it was shown that differences exist between identical specimens grown under different laboratory conditions (Mo et al., 2018). According to Peres et al. (2002), there is an overlap between the conidial morphology and cultural characteristics, thus making the use of these features unreliable.

Biochemical reactions and immunoassays have been used to distinguish between bacterial, fungal, protozoan, and plant species based on differences in certain structural features associated with these organisms. Some of these reactions have been employed to separate the different species of MAD causative agents. Honger et al. (2014) used the casein hydrolysis method to distinguish between *C. gloeosporioides* and *C. acutatum* in mango isolates from Ghana. Their study found that *C. gloeosporioides* and *C. acutatum* tested negative and positive, respectively for the casein hydrolysis method. This result further confirmed that the two species are different; hence, they concluded that the main causative species in Ghana was *C. gloeosporioides* and not *C. acutatum*.

Using indirect ELISA and western blotting techniques, Theerthagiri et al. (2016) could distinguish between the various causative agents of the disease. For the western blotting results, they found a 40 kDa molecular weight protein in *C. gloeosporioides* which was different from other *Colletotrichum* species. The indirect ELISA data gave higher titer values of polyclonal antibodies to protein extracts of *C. gloeosporioides* compared to other fungal species, further confirming that it differs from the other *Colletotrichum* species.

With technological advancement, molecular techniques have been employed to augment morphological and biochemical detection methods. Molecular techniques are accurate, rapid, specific, and sensitive in detecting the causative organisms of the disease and thus help in understanding the mechanism involved in disease pathology and management (Kamle et al., 2013). Molecular techniques have been developed based on species’ ribosomal DNA (rDNA) differences. The internal transcribed spacer (ITS) regions (ITS 1 and ITS 4) within the nuclear ribosomal gene cluster have mainly been found to be a good site for the design of specific primers for the detection of the causative agents of the disease (Freeman et al., 2000; Kamle et al., 2013; Zakaria et al., 2015). In a study to distinguish between *Colletotrichum* species, species-specific primers MKCgF and MKCgR of amplicon size 380 bp were designed. The study showed that the primers could amplify all isolates of *C. gloeosporioides* but not the other species, such as *C. acutatum*, *C. falcatum* and *C. capsici*. It was therefore suggested that the MKCgF and MKCgR would be a good marker for distinguishing between species-specific *Colletotrichum* and hence valuable for developing a rapid and sensitive a diagnostic PCR assay for early detection and management of the disease (Kamle et al., 2013). In another study from Ghana using 480 bp species-specific primers CgInt and ITS 4, it was observed that the *C. gloeosporioides* complex showed amplification products at 480 bp while *C. acutatum* did not. Phylogenetic analysis also showed that the *C. acutatum* clade clustered far away from the *C. gloeosporioides* complex, thus indicating that the two species are different (Honger et al., 2014).

Although the ITS gene sequence is instrumental in identifying *Colletotrichum* species, it cannot distinguish between closely related species (Cannon et al., 2012). This, therefore, has led to the use of multiple gene sequences to differentiate between and within species (Weir et al., 2012; Dela Cueva et al., 2021). Due to the drawback of using ITS, other genes such as actin, β – tubulin, chitin synthase, glyceraldehyde-3-phosphate dehydrogenase, calmodulin, and glutamine synthetase have also been studied to explore the differences among *Colletotrichum* species (Weir et al., 2012; Lima et al., 2013a,b; Dela Cueva et al., 2021). Lima et al. (2013a,b), in their study to identify the *Colletotrichum* species associated with MAD in northeastern Brazil, used sets of primers for the glyceraldehyde-3-phosphate, actin, β – tubulin, calmodulin, and glutamine synthetase genes. It was found that the primers were able to amplify five different species from the *C. gloeosporioides* complex namely *C. asianum*, *C. fructicola*, *C. tropicale*, *C. karstii*, and *C. dianesei*. A similar result was obtained in mango leaves in which *C. asianum*, *C. fructicola* and *C. siamense* were identified using glyceraldehyde-3-phosphate dehydrogenase, partial actin, β – tubulin, and chitin synthase (Mo et al., 2018). Therefore, the findings suggest that these genes could be used to detect different strains within a species complex.

Current methods focus on designing and developing imaging processing and algorithms that have aided in automated disease detection. Accurate techniques such as camera-assisted image analysis (Corkidi et al., 2006) and a computer vision system based on ultraviolet light (Alberto et al., 2022) have been employed in the visual detection of the disease. However, this is only effective if the symptoms appear on the skin of the fruits hence unable to detect the disease at an early stage. A hyperspectral imaging spectrum based on spectroscopy and computer vision has been developed for early detection of the disease. The technique has been shown to provide information about the spatial distribution of components in the mango plant for easy detection and classification under different infection levels (Siriptrawan, 2021). Another computer study was done using a Matrix Laboratory (MATLAB) based disease detection system. The system employs a Gray Level Co-occurrence Matrix (GLCM) algorithm, which can extract the features of the disease, and an SVM classifier that classifies the disease type based on the extracted features from the GLCM. The study observed that the system yields a 90% accuracy of automated detection and provides appropriate preventive and curative solutions (Veling et al., 2019). Thus, a combination of different detection methods can ensure efficient and accurate disease detection.

## 9. Chemical and host plant resistance

In Taiwan, systemic fungicides known as Benzimidazoles are used to control *C. gloeosporioides* (Chung et al., 2010). Certain strains of this fungus show different levels of resistance to Benzimidazole. There are known *C. gloeosporioides* isolates from resistant crops, but we still need a complete picture of their molecular features (Chung et al., 2010). Benzimidazole-resistant strains have been found in Japan (Tashiro et al., 2012). There appears to be a spectrum of resistance among the species in the *C. gloeosporioides* Species Complex (CGSC), as determined by genetic analysis (Yokosawa et al., 2017). It has been reported that benomyl-resistant *C. gloeosporioides* f.sp. *malvae* was isolated from uv-irradiated, actively developing mycelium (Holmström-Ruddick and Mortensen, 1995). Mycoherbicide agents for round-leaved mallow can be registered for this plant. Benomyl foliar spray has been shown to be quite effective in preventing the spread of *C. gloeosporioides*; nevertheless, some strains have become resistant to the spray after repeated administration. The amino acid sequence at the benzimidazole binding site can change because of a point mutation in a β-tubulin gene resulting in fungal resistance to benomyl (Maymon et al., 2006). Two β-tubulin genes have been characterized in some *Colletotrichum* spp., namely TUB1 and TUB2 (Maymon et al., 2006).

*Colletotrichum gloeosporioides* has become a global problem causing significant economic damage (Chung et al., 2010) with some strains being resistant and difficult to control using specific chemicals. The microorganism has been classified taxonomically using microscopic and morphological characteristics. Resistance strains are microorganisms that do not yield to the expected effect of chemicals. Some phenolic lipids in living organisms include alkyl phenols, alkyl resorcinols, anarcadic acids, and alkyl catechols (Supriya et al., 2020). Alkylresorcinols impart resistance to plants and other living organisms against abiotic and biotic stresses (Supriya et al., 2020). They do not only elicit defensive action in plants grown in biotic stress conditions, but the alkylresorcinols extracted from rye can inhibit the mycelial growth of *Fusarium culmorum* and *Rhizoctonia solani.* There are also reports of resistance by *C. gloeosporioides* to systemic fungicides named Benzimidazoles (Chung et al., 2010). Different strains have different levels of pathogenicity to different plants (Sanchez-Arizpe et al., 2021).

Natural disease resistance in Mango varieties is being exploited as a control against anthracnose disease in mango fruits (Gong et al., 2013). Different cultivars show different levels of disease resistance. A study was carried out on the resistance of two mango varieties (‘Keitt’ and ‘Zill’) to anthracnose disease. *C. gloeosporioides* infection of young or commercially ripe fruit resulted in reduced lesion diameters on ‘Keitt’ fruit compared to ‘Zill’ fruit (Gong et al., 2013). When non-inoculated fruits were harvested at commercial maturity, “Keitt” showed a lower disease index than ‘Zill’, indicating that ‘Keitt’ was more disease resistant than ‘Zill’ (Gong et al., 2013). More significant amounts of hydrogen peroxide, total phenolic compounds, and lignin were found in ‘Keitt’ fruit during development and storage compared to ‘Zill’ fruit. Additionally, ‘Keitt’ fruit had higher contents of hydrogen peroxide and lignin in harvested fruit early during storage (Gong et al., 2013). These findings highlight the importance of defense enzymes and chemicals in mango fruit’s resistance to MAD. It has been proposed that these could be utilized as markers to screen for mango cultivars with increased resistance to post-harvest illnesses (Gong et al., 2013).

Resistant varieties vary in their ability to suppress disease development in the crop. The varieties may have specific biochemical differences which enable them to suppress disease development, and humanity can exploit the suppressive ability to overcome the problems of resistant strains. In order to understand these biochemical differences, a study was conducted in India to find the constitutive antifungal phenolic lipids, phenolics contents, and antioxidant activities in a resistant variety of mango known as ‘Kensingtonpride’ and two susceptible varieties known as ‘Badami’ and ‘Raspuri’; their resistance and susceptibility were to the MAD (Supriya et al., 2020). Both the 5-n-pentadecyl resorcinol and total phenolics levels were measured using phytochemical analysis. Furthermore, the antioxidant potential of the mango peel methanolic extracts was evaluated using *in vitro* DPPH assay. The results demonstrate that early in fruit growth (at 30DFS), ‘Kensington pride,’ as opposed to ‘Badami’ and ‘Raspuri,’ has the highest concentration of 5-n-pentadecyl resorcinol in the mango fruit peel extract. High levels (*p* ≤ 0.05) of constitutive antifungal 5-n-pentadecyl resorcinol in anthracnose-resistant mango cultivar (‘Kensington pride’) was endogenously produced and retained during the early stage of fruit development. During plants’ growing, maturing, and ripening stages, this molecule confers resistance to disease, and it may serve as a foundation of the plant’s defense mechanism against anthracnose (Supriya et al., 2020).

The resistant varieties of mango contain fungi toxic levels of antifungal resorcinols, which enable the mangoes to resist infection (Karunanayake et al., 2014). In China, it has been shown that by manipulating temperatures and carbendazim concentrations, the strains of *C. gloesporioides* resistant to Benzamidazole can be effectively controlled (Lin et al., 2016). Screening of mango hybrids against the disease is also a step taken by several research institutes (Bally et al., 2010). It is advisable to use fungicides to which the strains are not resistant. Mancozeb and Copper Oxychloride are two fungicides currently being used, and the latter is known to increase fruit set (Uddin et al., 2018).

## 10. Management of the disease

Management plans must be cost-effective, efficient, and safe for the environment, consumers, and agricultural workers. Pre-harvest, postharvest, or, ideally, a mix of both treatments are effective ways to control anthracnose. A wide range of options are available for dealing with MAD in the field. Fungicide spraying, proper cultural techniques, and the choice of cultivars are the primary components.

The varied responses of cultivars to the disease in various regions have prevented resistance from being consistently employed as a method of controlling MAD. With an appropriate mango variety, growers can grow a crop with fewer incidences of anthracnose, resulting in an increased yield and fruit of superior quality. Resistance also reduces the need for fungicides to protect the crop, making it more cost-effective and sustainable (Leadbeater, 2014).

Wet conditions or high relative humidity are necessary for the disease to flourish (Božič and Kanduč, 2021). Therefore, locating farms in areas with a distinct dry season is ideal, as this promotes disease-free fruit growth. Additionally, it has been suggested that field sanitation procedures incorporate collecting and burning trash from fallen trees and fruit (Sosnowski et al., 2009).

Fungicide use has received the majority of the focus and attention in the fight against anthracnose. Inflorescence and fruit damage are minimized with the administration of fungicides. Different fungicides may be utilized depending on where the exported fruit will go. Non-systemic fungicides like zineb, maneb, or captan, applied weekly during blossoming and then monthly during fruit development, were shown in early experiments to be effective against the disease (Sardrood and Goltapeh, 2018). Applying copper oxychloride or copper oxychloride in conjunction with zineb in pre-harvest management is recommended for anthracnose control in South Africa every 14 days in wet conditions and every 28 days in dry conditions (Akem, 2006). In addition, after the fruit had begun to set, mancozeb and copper oxychloride were alternated every month until harvest. Although copper fungicides are recommended, they are not as effective as dithiocarbamates when dealing with high disease pressure and hence pose a worry for phytotoxicity on mango blossoms (Thind and Hollomon, 2018). Fungicides with an after-infection activity, such as benzimidazoles and imidazole prochloraz, are effective against MAD. Scheduled applications of benomyl, sometimes in conjunction with protectant fungicides, have been used to halt the development of resistance in pathogen populations. Both preventative and curative applications of prochloraz have been made (Chiangsin et al., 2016).

Prioritizing the reduction of dormant infections on mangoes could be a mainstay of postharvest management strategies for MAD. Anthracnose in ripe fruit has been a target of postharvest treatments for a long time. It has been shown that hot water dips are useful for controlling anthracnose (Mirshekari et al., 2012). Commercially, latent infection is removed using heat, chemicals, or a mix of both approaches. It has been suggested to expose subjects to temperatures between 50 and 55°C for exposure durations ranging from 3 to 15 min (Mirshekari et al., 2012). To control anthracnose, a variety of fungicides have been used after harvest. Prochloraz and Thiabendazole can be utilized. However, their effectiveness varies with the severity of the condition (Shi et al., 2020). One benefit of benzimidazole fungicides like benomyl or thiabendazole is that they work well to suppress stem-end rot on mangoes brought on by *Lasiodiplodia theobromae* (Pat.), which is regarded as the second most significant postharvest disease of mango in tropical regions (Shi et al., 2020). Postharvest techniques, including a controlled environment and cold storage, maintain resistance to deterioration by postponing the ripening phase. The potential benefit of this strategy has several restrictions. Mangoes suffer damage at temperatures below 10 to 13°C because they are susceptible to chilling (Patel et al., 2016). Disease naturally develops if the fruit is allowed to ripe in natural settings. There have been limited and inconsistent attempts at postharvest biological control of MAD. Numerous microorganisms have been implicated in the cases of MAD as having antagonistic relationships with the disease’s causal agent (*C. gloeosporioides*) (Kankam et al., 2022). For example, *Trichoderma* spp. *Bacillus* spp. and *Pseudomonas* spp. are known for their antagonistic properties against plant pathogens (Zin and Badaluddin, 2020). They have been reported to be effective in reducing the incidence of MAD by competing with *C. gloeosporioides* for nutrients and space. However, there is currently no evidence of this finding’s commercial implementation, although it appears to be quite promising.

Recent studies have also revealed the success of using botanicals to cure MAD (Alemu et al., 2014). The *in vitro* mycelial growth of *C. gloeosporioides* has been demonstrated to be suppressed by aqueous extracts of the leaves of *Eucalyptus camaldulensis* and *Azadirachta indica*. Anthracnose on mango trees was reduced in both occurrence and severity after foliar application of the extracts, even in field settings (Haider et al., 2020). When applied to mangoes before storage, aqueous extracts of *Ruta chalepensis* at a concentration of 50 grams of the powdered plant material in 100 mL of distilled water effectively decreased the incidence of anthracnose disease, maintaining quality, and making the fruits more marketable (Alemu et al., 2014).

**Figure 4:**
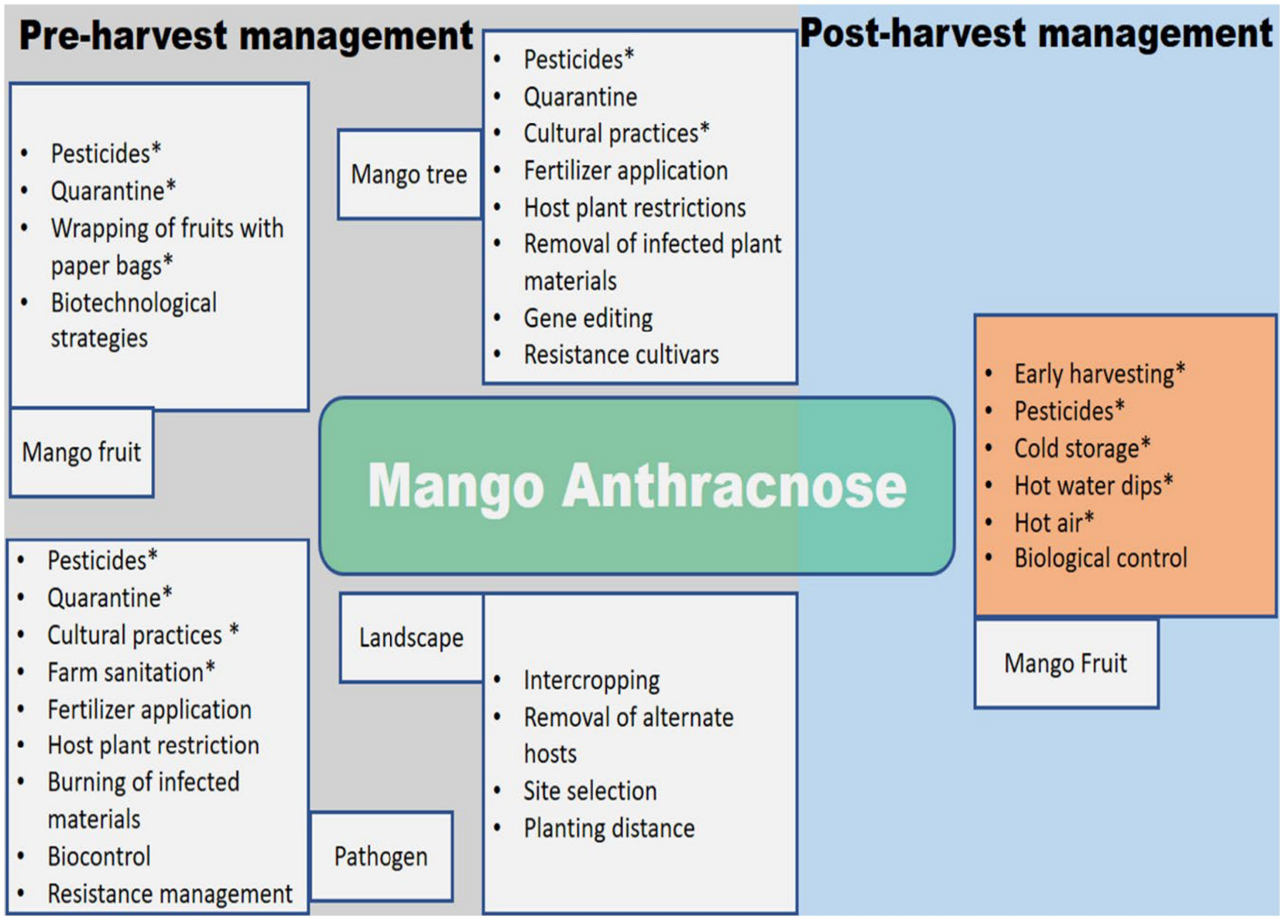
Schematic diagram for the management of mango anthracnose. Options that have had an influence in at least some field settings are indicated by an asterisk (*).

The inadequacies and inconsistent efficacy of postharvest treatments demonstrate the need for an integrated strategy in managing MAD. Figure 4 provides a schematic illustration of the various management strategies employed to deal with MAD before and after harvest.

## 11. Conclusion and future prospects

MAD is still a widespread fungal disease that lowers production and quality worldwide. *Colletotrichum* is endemic to nearly all places where mangoes are grown, posing a significant threat to the industry worldwide. Decay patches from dark brown to black appear on leaves, twigs, flowers, and fruits during the pre-and postharvest stages of the disease. The pathogenic fungus that causes MAD tends to infest unripe mango fruits and become dormant until the fruit ripens. The extensive use of synthetic fungicides to prevent the occurrences of MAD can lead to environmental pollution, water pollution, soil compaction, and ecological problems, which then affect the sustainability of mango production. Research on the biological characteristics and behavior of the pathogen, epidemiology, and management of MAD has recently increased. The availability of genomic data on *C*. *gloeosporioides* has provided a better understanding and knowledge of the host-fungal interaction. This has also assisted in developing new methods for rapidly detecting *C*. *gloeosporioides*.

Significant progress in MAD management has been made, especially concerning fungicides and timing treatments, postharvest treatments, and resistant cultivars. However, improper identification of closely related species of the pathogen that causes MAD remains a significant challenge in managing the disease. This has been attributed to the similarities in the morphological traits of pathogens and their symptoms on the host plant. Plant breeding projects for developing new disease-resistant mango cultivars could benefit from a more in-depth understanding of the disease’s epidemiology. There is little known about *C. gloeosporioides’* population structure, genetic diversity, or sensitivity to varying fungicide concentrations. Further research on the population, diversity, and fungicides sensitivity of the pathogen is vital for developing effective management strategies for the disease.

MAD remains the most crucial economic disease worldwide in almost all mango production areas. It requires pre-harvest and postharvest strategies for effective control because it affects nearly all of the plant’s above-ground portions. Timely application of appropriate fungicides in the field, good cultural practices, resistant cultivars, and appropriate postharvest treatments, such as dip treatments and refrigeration, are only some of the management methods that have been called for to lessen the impact of this threat. However, picking the best control approach requires more information about this condition and public awareness.

Alternatives to fungicides have been developed in light of rising awareness of the fungicides’ deleterious effects on human health, the presence of fungicide residues in mango fruits, and the contamination of the natural environment that results from their widespread use. Essential oils, botanicals, and oxalic acid treatments have all been shown to be effective alternatives to chemical control, especially in locations where the use of synthetic fungicides is prohibited. Microbial agents and biological control of the MAD have also broadened developmental prospects for establishing environmentally friendly pest management.

Furthermore, resistance breeding is vital to the control of MAD. Research on host-pathogen interaction and identification of fungal genes underlying virulence, phytotoxins of *C*. *gloeosporioides*, and its pathway genes will improve knowledge of resistance mechanisms and management of the disease. Invading fungi usually secrete plant growth regulators such as auxin, cytokines, ethylene, gibberellic acid, jasmonic acid, and salicylic acid. These growth regulators distract plants’ levels of endogenous hormones, weakening their defense mechanism against the pathogenic fungus. More targets for the development of innovations or fungicides against *C. gloeosporioides* can be found through a systematic investigation of the manufacturing and signal transduction pathways of pathogenic fungal hormones.

Pathogenic fungi also release effector proteins, which serve crucial functions in plant cells and modify the interaction between pathogens and their hosts. Little is known about the fungal effectors that cause mango disease. The host-plant relationship, the pathogenic fungus’s pathogenic processes, and host plants’ disease-resistance mechanisms may all benefit from functional investigations and comparative analyses of the *C. gloeosporioides* effector protein.

The key motivating factors for creating alternative techniques to reduce MAD include the recent understanding of health dangers, the rising customer preference for healthy agricultural products, and the environmental pollution connected with fungicide usage. Further research should be encouraged on host-pathogenic fungal interaction, transmission, and effective control techniques. Shortly, research on MAD should be geared toward developing techniques that address many environmental factors and pathogenic fungal resistance. Future research on developing and deploying electronic and disease sensors for rapid detection of *C*. *gloeosporioides* on-site and entry points of disease-free regions are recommended to minimize the spread of MAD.

For instance, smartphone-based fingerprinting of leaf volatiles has may be to detect the late blight of tomatoes. In addition, a microneedle patch coupled with a loop-meditated isothermal amplification-based sensor may be developed to detect coinfections of late blight and spotted wilt virus in tomatoes. These strategies could be adopted for MAD and a more comprehensive and coordinated surveillance and monitoring strategies for MAD that includes all stakeholders, government, and non-government organizations would help to reduce the migration of the disease pathogens.

Furthermore, research programs that focus on disease surveillance through risk modeling, bioinformatics tools, and geospatial analytic tools for mapping and analyzing data about the pathogen and its host must be deployed to respond to potential anthracnose threats in mango production. Global sharing of data or information by researchers and policymakers, and identification of a global hot spot for new outbreaks of MAD will be needed to manage the disease. There is still more to learn about MAD, its detection, and causal organism and management strategies. Future research areas should include; how climate change affects the spread and management of MAD, sensors for on-site detection, and the deployment of gene editing, nanoparticles, and nanotechnology for managing the diseases.

## Author contributions

AD and OA: study-conceived and designed, writing–original draft, review, and editing. NQ, AO, AA-A, BB, FA, KA, HL, SL, JO-O, JO, WE, and JH: writing–original draft. KN: review and editing. All authors approved the submitted version.

## Conflict of interest

The authors declare that the research was conducted in the absence of any commercial or financial relationships that could be construed as a potential conflict of interest.

## Publisher’s note

All claims expressed in this article are solely those of the authors and do not necessarily represent those of their affiliated organizations, or those of the publisher, the editors and the reviewers. Any product that may be evaluated in this article, or claim that may be made by its manufacturer, is not guaranteed or endorsed by the publisher.

## References

[ref1] Abang M. M. Winter S. Green K. R. Hoffmann P. Mignouna H. D. Wolf G. A. (2002). Molecular identification of *Colletotrichum gloeosporioides* causing yam anthracnose in Nigeria. Plant Pathol. 51, 63–71. doi: 10.1046/j.0032-0862.2001.00655.x

[ref2] Abera A. Lemessa F. Adunga G. (2016). Morphological characteristics of *Colletotrichum* species associated with mango (*Mangifera indica* L.) in Southwest Ethiopia. Food Sci. Qual. Manag. 48, 106–115.

[ref3] Ahmad T. Wang J. Zheng Y. Mugizi A. E. Moosa A. Nie C.. (2021). First record of *Colletotrichum alienum* causing postharvest anthracnose disease of mango fruit in China. Plant Dis. 105:1852. doi: 10.1094/PDIS-09-20-2074-PDN

[ref4] Ajitomi A. Takushi T. Sato Y. Arasaki C. Ooshiro A. (2020). First report of powdery mildew of mango caused by *Erysiphe quercicola* in Japan. J. Gen. Plant Pathol. 86, 316–321. doi: 10.1007/s10327-020-00918-2

[ref5] Akem C. N. (2006). Mango anthracnose disease: present status and future research priorities. Plant Pathol. J. 5, 266–273. doi: 10.3923/ppj.2006.266.273

[ref6] Alberto L. R. Ardila C. E. C. Ortiz F. A. P. (2022). A computer vision system for early detection of anthracnose in sugar mango (*Mangifera indica*) based on UV-A illumination. Inf. Process. Agric. 10, 204–215. doi: 10.1016/j.inpa.2022.02.001

[ref7] Alemu K. Ayalew A. Woldetsadik K. (2014). Antifungal activity of plant extracts and their applicability in extending the shelf-life of mango fruits. Food Sci. Qual. Manag. 33, 47–53.

[ref8] Alkan N. Meng X. Friedlander G. Reuveni E. Sukno S. Sherman A.. (2013). Global aspects of pacC regulation of pathogenicity genes in *Colletotrichum gloeosporioides* as revealed by transcriptome analysis. Mol. Plant-Microbe Interact. 26, 1345–1358. doi: 10.1094/MPMI-03-13-0080-R 23902260

[ref9] Anderson J. M. Aitken E. A. B. Dann E. K. Coates L. M. (2013). Morphological and molecular diversity of *Colletotrichum* spp. causing pepper spot and anthracnose of lychee (*Litchi chinensis*) in Australia. Plant Pathol. 62, 279–288. doi: 10.1111/j.1365-3059.2012.02632.x

[ref10] Arauz L. F. (2000). Mango anthracnose: economic impact and current options for integrated managaement. Plant Dis. 84, 600–611. doi: 10.1094/PDIS.2000.84.6.600, PMID: 30841097

[ref11] Ashraful A. Sanjoy K. A. Mahtalat A. (2017). Morphological characterization of *Colletotrichum gloeosporioiedes* identified from anthracnose of *Mangifera indica* L. Asian J. Plant. Pathol. 11, 102–117. doi: 10.3923/ajppaj.2017.102.117

[ref12] Awa O. C. Samuel O. Oworu O. O. Sosanya O. (2012). First report of fruit anthracnose in mango caused by *Colletotrichum gloeosporioides* in southwestern Nigeria. Int. J. Sci. Technol. Res. 1, 30–34.

[ref13] Bally I. S. Akem C. N. Dillon N. L. Grice C. Lakhesar D. Stockdale K. (2010). Screening and breeding for genetic resistance to anthracnose in mango. In IX International Mango Symposium 992 (pp. 239–244)

[ref14] Barhoom S. Kupiec M. Zhao X. Xu J. R. Sharon A. (2008). Functional characterization of CgCTR2, a putative vacuole copper transporter that is involved in germination and pathogenicity in *Colletotrichum gloeosporioides*. Eukaryot. Cell 7, 1098–1108. doi: 10.1128/EC.00109-07, PMID: 18456860 PMC2446676

[ref15] Barhoom S. Sharon A. (2007). Bcl-2 proteins link programmed cell death with growth and morphogenetic adaptations in the fungal plant pathogen *Colletotrichum gloeosporioides*. Fungal Genet. Biol. 44, 32–43. doi: 10.1016/j.fgb.2006.06.007, PMID: 16950636

[ref16] Benatar G. V. Wibowo A. Suryanti (2021). First report of *Colletotrichum asianum* associated with mango fruit anthracnose in Indonesia. Crop Prot. 141:105432. doi: 10.1016/j.cropro.2020.105432

[ref17] Bhagwat R. G. Mehta B. P. Patil V. A. Sharma H. (2015). Screening of cultivars/varieties against mango anthracnose caused by *Colletotrichum gloeosporioides*. Int. J. Environ. Agric. Res. 1, 21–23.

[ref18] Bordoh P. K. Ali A. Dickinson M. Siddiqui Y. Romanazzi G. (2020). A review on the management of postharvest anthracnose in dragon fruits caused by *Colletotrichum* spp. Crop Prot. 130:105067. doi: 10.1016/j.cropro.2019.105067

[ref19] Božič A. Kanduč M. (2021). Relative humidity in droplet and airborne transmission of disease. J. Biol. Phys. 47, 1–29. doi: 10.1007/s10867-020-09562-5, PMID: 33564965 PMC7872882

[ref20] Caicedo J. D. Lalangui K. P. Pozo A. N. Cevallos P. A. Arahana V. S. Méndez K. S. (2017). Multilocus molecular identification and phylogenetic analysis of *Colletotrichum tamarilloi* as the causal agent of Tamarillo (*Solanum betaceum*) anthracnose in the Ecuadorian highlands. Europ. J. Plant Pathol. 148, 983–996. doi: 10.1007/s10658-017-1155-3

[ref21] Cannon P. F. Damm U. Johnston P. R. Weir B. S. (2012). *Colletotrichum*: current status and future directions. Stud. Mycol. 73, 181–213. doi: 10.3114/sim0014, PMID: 23136460 PMC3458418

[ref22] Cao X. R. Xu X. M. Che H. Y. West J. S. Luo D. Q. (2019). Characteristics and distribution of *Colletotrichum* species in coffee plantations in Hainan, China. Plant Pathol. 68, 1146–1156. doi: 10.1111/ppa.13028

[ref23] Castillo S. R. M. Miller S. Stewart J. (2022). *Colletotrichum* spp. and other fungi associated with anthracnose on *Coffea arabica* L. in Mérida state, Venezuela. Summa Phytopathol. 48, 99–111. doi: 10.1590/0100-5405/245876

[ref24] Chagué V. Maor R. Sharon A. (2009). CgOpt1, a putative oligopeptide transporter from *Colletotrichum gloeosporioides* that is involved in responses to auxin and pathogenicity. BMC Microbiol. 9, 1–12. doi: 10.1186/1471-2180-9-173, PMID: 19698103 PMC2769210

[ref25] Chala A. Getahun M. Alemayehu S. Tadesse M. (2014). Survey of mango anthracnose in southern Ethiopia and *in-vitro* screening of some essential oils against *Colletotrichum gloeosporioides*. Int. J. Fruit Sci. 14, 157–173. doi: 10.1080/15538362.2013.817899

[ref26] Cheng Y. J. Wu Y. J. Lee F. W. Ou L. Y. Chen C. N. Chu Y. Y.. (2022). Impact of storage condition on chemical composition and antifungal activity of pomelo extract against *Colletotrichum gloeosporioides* and anthracnose in post-harvest mango. Plan. Theory 11:2064. doi: 10.3390/plants11152064, PMID: 35956542 PMC9370353

[ref27] Chiangsin R. Wanichkul K. Guest D. I. Sangchote S. (2016). Reduction of anthracnose on ripened mango fruits by chemicals, fruit bagging, and postharvest treatments. Australas. Plant Pathol. 45, 629–635. doi: 10.1007/s13313-016-0456-x

[ref28] Chowdhury M. N. Rahim M. A. (2009). Integrated crop management to control anthracnose (*Colletotrichum gloeosporioides*) of mango. J. Agric. Ext. Rural Dev., 115–120. doi: 10.3329/jard.v7i1.4430

[ref29] Chung W. H. Chung W. C. Peng M. T. Yang H. R. Huang J. W. (2010). Specific detection of benzimidazole resistance in *Colletotrichum gloeosporioides* from fruit crops by PCR-RFLP. New Biotechnol. 27, 17–24. doi: 10.1016/j.nbt.2009.10.004, PMID: 19854306

[ref30] Ciofini A. Negrini F. Baroncelli R. Baraldi E. (2022). Management of Post-Harvest Anthracnose: current approaches and future perspectives. Plan. Theory 11:1856. doi: 10.3390/plants11141856, PMID: 35890490 PMC9319655

[ref31] Corda A. C. I. (1831). “Die Pilze Deutschlands” in Deutschlands Flora in Abbildungen nach der Natur mit Beschreibungen. ed. Sturm J., vol. 3 (Nürnberg: Sturm), 33–64.

[ref32] Corkidi G. Balderas-Ruíz K. A. Taboada B. Serrano-Carreón L. Galindo E. (2006). Assessing mango anthracnose using a new three-dimensional image-analysis technique to quantify lesions on fruit. Plant Pathol. 55, 250–257. doi: 10.111/j.1365-3059.2005.01321.x

[ref33] Costa J. F. Kamei S. H. Silva J. R. A. Miranda A. R. G. D. S. Netto M. B. da Silva S. J. C.. (2019). Species diversity of *Colletotrichum* infecting Annona spp. in Brazil. Europ. J. Plant Pathol. 153, 1119–1130. doi: 10.1007/s10658-018-01630-w

[ref34] Cristóbal-Martínez A. L. de Jesús Yáñez-Morales M. Solano-Vidal R. Segura-León O. Hernández-Anguiano A. M. (2017). Diversity of *Colletotrichum* species in coffee (*Coffea arabica*) plantations in Mexico. Eur. J. Plant Pathol. 147, 605–614. doi: 10.1007/s10658-016-1029-0

[ref35] Damm U. Cannon P. F. Woudenberg J. H. C. Crous P. W. (2012). The *Colletotrichum acutatum* species complex. Stud. Mycol. 73, 37–113. doi: 10.3114/sim0010, PMID: 23136458 PMC3458416

[ref36] De Jonge R. Peter van Esse H. Kombrink A. Shinya T. Desaki Y. Bours R.. (2010). Conserved fungal LysM effector Ecp6 prevents chitin-triggered immunity in plants. Science 329, 953–955. doi: 10.1126/science.1190859, PMID: 20724636

[ref37] De Souza A. Delphino Carboni R. C. Wickert E. de Macedo Lemos E. G. de Goes A. (2013). Lack of host specificity of *Colletotrichum* spp. isolates associated with anthracnose symptoms on mango in Brazil. Plant Pathol. 62, 1038–1047. doi: 10.1111/ppa.12021

[ref38] Dela Cueva F. M. Laurel N. R. Dalisay T. U. Sison M. L. J. (2021). Identification and characterisation of *Colletotrichum fructicola*, *C. tropicale* and *C. theobromicola* causing mango anthracnose in the Philippines. Arch. Phytopathol. 54, 1989–2006. doi: 10.1080/03235408.2021.1968234

[ref39] Dembele D. D. Camara B. Grechi I. Rey J. Y. Kone D. (2019). Pre and postharvest assessment of mango anthracnose incidence and severity in the north of Côte d’Ivoire. Int. J. Biol. Chem. Sci. 10, 33–43. doi: 10.3923/IJAR.2015.33.43

[ref40] Doyle V. P. Oudemans P. V. Rehner S. A. Litt A. (2013). Habitat and host indicate lineage identity in *Colletotrichum gloeosporioides* sl from wild and agricultural landscapes in North America. PLoS One 8:e62394. doi: 10.1371/journal.pone.0062394, PMID: 23671594 PMC3646003

[ref41] Drori N. Kramer-Haimovich H. Rollins J. Dinoor A. Okon Y. Pines O.. (2003). External pH and nitrogen source affect secretion of pectate lyase by *Colletotrichum gloeosporioides*. Appl. Environ. Microbiol. 69, 3258–3262. doi: 10.1128/AEM.69.6.3258-3262.2003, PMID: 12788724 PMC161482

[ref42] Evangelista-Martínez Z. Ek-Cen A. Torres-Calzada C. Uc-Várguez A. (2022). Potential of *Streptomyces* sp. strain AGS-58 in controlling anthracnose-causing *Colletotrichum siamense* from post-harvest mango fruits. J. Plant Pathol. 104, 553–563. doi: 10.1007/s42161-022-01104-3

[ref43] Feng G. Zhang X. S. Zhang Z. K. Ye H. C. Liu Y. Q. Yang G. Z.. (2019). Fungicidal activities of camptothecin semisynthetic derivatives against *Colletotrichum gloeosporioides* in vitro and in mango fruit. Postharvest Biol. Technol. 147, 139–147. doi: 10.1016/j.postharvbio.2018.09.019

[ref44] Freeman S. Minz D. Jurkevitch E. Maymon M. Shabi E. (2000). Molecular analyses of *Colletotrichum* species from almond and other fruits. Phytopathology 90, 608–614. doi: 10.1094/PHYTO.2000.90.6.608, PMID: 18944540

[ref45] Fujikawa T. Sakaguchi A. Nishizawa Y. Kouzai Y. Minami E. Yano S.. (2012). Surface α-1, 3-glucan facilitates fungal stealth infection by interfering with innate immunity in plants. PLoS Pathog. 8:e1002882. doi: 10.1371/journal.ppat.1002882, PMID: 22927818 PMC3426526

[ref46] Gan P. Ikeda K. Irieda H. Narusaka M. O'Connell R. J. Narusaka Y.. (2013). Comparative genomic and transcriptomic analyses reveal the hemibiotrophic stage shift of *Colletotrichum* fungi. New Phytol. 197, 1236–1249. doi: 10.1111/nph.12085, PMID: 23252678

[ref47] Gautam C. Prabhu H. V. Nargund V. B. (2017). *In vitro* evaluation of fungicides, botanicals and bioagents against *Peziotrichum corticolum* causing black banded disease of mango. Int. J. Curr. Microbiol. Appl. Sci. 6, 652–661. doi: 10.20546/ijcmas.2017.603.076

[ref48] Gong D. Q. Zhu S. J. Gu H. Zhang L. B. Hong K. Q. Xie J. H. (2013). Disease resistance of ‘Zill’and ‘Keitt’mango fruit to anthracnose in relation to defence enzyme activities and the content of anti-fungal substances. J. Hortic. Sci. Biotechnol. 88, 243–250. doi: 10.1080/14620316.2013.11512962

[ref49] Grammen A. Wenneker M. Van Campenhout J. Pham K. T. K. Van Hemelrijck W. Bylemans D.. (2019). Identification and pathogenicity assessment of *Colletotrichum* isolates causing bitter rot of apple fruit in Belgium. Eur. J. Plant Pathol. 153, 47–63. doi: 10.1007/s10658-018-1539-z

[ref50] Grice K. R. E. Bally I. S. E. Wright C. L. Maddox C. Ali A. Dillon N. L. (2022). Mango germplasm screening for the identification of sources of tolerance to anthracnose. Australas. Plant Pathol. 52, 27–41. doi: 10.1007/s13313-022-00899-0

[ref51] Guevara-Suarez M. Cárdenas M. Jiménez P. Afanador-Kafuri L. Restrepo S. (2022). *Colletotrichum* species complexes associated with crops in northern South America: a review. Agronomy 12:548. doi: 10.3390/agronomy12030548

[ref52] Haider E. Khan M. A. Atiq M. Shahbaz M. Yaseen S. (2020). Phytoextracts as management tool against fungal diseases of vegetables. Int. J. Biosci. 16, 303–314. doi: 10.12692/ijb/16.3.303-314

[ref53] Hofer K. M. Braithwaite M. Braithwaite L. J. Sorensen S. Siebert B. Pather V.. (2021). First report of *Colletotrichum fructicola*, *C. perseae*, and *C. siamense* causing anthracnose disease of avocado (*Persea americana*) in New Zealand. Plant Dis. 105:1564. doi: 10.1094/PDIS-06-20-1313-PDN

[ref54] Holmström-Ruddick B. Mortensen K. (1995). Factors affecting pathogenicity of a benomyl-resistant strain of *Colletotrichum gloeosporioides* f. sp. malvae. Mycol. Res. 99, 1108–1112. doi: 10.1016/S0953-7562(09)80780-6

[ref55] Honger J. O. Offei S. K. Oduro K. A. Odamtten G. T. (2015). Nature of mango anthracnose in Ghana: implications for the control of the disease. Ghana J. Agric. Sci. 49, 53–67.

[ref56] Honger J. O. Offei S. K. Oduro K. A. Odamtten G. T. Nyaku S. T. (2014). Identification and species status of the mango biotype of *Colletotrichum gloeosporioides* in Ghana. Eur. J. Plant Pathol. 140, 455–467. doi: 10.1007/s10658-014-0480-z

[ref57] Hossain A. A. Ahmed A. (1994). A monograph on mango varieties of Bangladesh. Dhaka: Bangladesh Agricultural Research Institute.

[ref58] Ismail A. M. Cirvilleri G. Yaseen T. Epifani F. Perrone G. Polizzi G. (2015). Characterisation of *Colletotrichum* species causing anthracnose disease of mango in Italy. J. Plant Pathol., 167–171. doi: 10.4454/JPP.V97I1.011

[ref59] Ismail A. M. El-Ganainy S. M. (2022). Characterization of *Colletotrichum* species associating with anthracnose disease of mango in Egypt. J. Plant Dis. Prot. 129, 449–454. doi: 10.1007/s41348-021-00538-8

[ref60] Janamatti A. T. Kumar A. Kaur C. Gogoi R. Varghese E. Kumar S. (2022). Fumigation by bacterial volatile 2, 5-dimethylpyrazine enhances anthracnose resistance and shelf life of mango. Eur. J. Plant Pathol. 164, 209–227. doi: 10.1007/s10658-022-02551-5

[ref61] Jenny F. Sultana N. Islam M. Khandaker M. M. Bhuiyan M. A. B. (2019). A review on anthracnose of mango caused by *Colletotrichum gloeosporioides*. Bangladesh J. Plant Phytopathol. 35, 65–74.

[ref62] Kadam J. A. Shimpi S. B. Biradar R. P. Chate S. V. (2002). Review on post harvest diseases and management of mango fruits. J. Plant Pathol. Microbiol. 13:1000635.

[ref63] Kamle M. Kumar P. (2016). *Colletotrichum gloeosporioides*: pathogen of anthracnose disease in mango (*Mangifera indica* L.). Curr. Trends Plant Dis. Diagn. Manag. Pract., 207–219. doi: 10.1007/978-3-319-27312-9_9

[ref64] Kamle M. Pandey B. K. Kumar P. Kumar M. (2013). A species-specific PCR based assay for rapid detection of mango anthracnose pathogen *Colletotrichum gloeosporioides* Penz. And Sacc. J. Plant Pathol. Microbiol. 4:184. doi: 10.4172/2157-7471.1000184

[ref65] Kankam F. Larbi-Koranteng S. Adomako J. Kwodaga J. K. Akpatsu I. B. Danso Y.. (2022). “Anthracnose disease of mango: epidemiology, impact and management options” in Current and emerging challenges in the diseases of trees (London: IntechOpen)

[ref66] Karunanayake K. O. L. C. Sinniah G. D. Adikaram N. K. B. Abayasekara C. L. (2014). Cultivar differences in antifungal activity and the resistance to postharvest anthracnose and stem-end rot in mango (*Mangifera indica* L.). Australas. Plant Pathol. 43, 151–159. doi: 10.1007/s13313-013-0257-4

[ref67] Khan M. S. Uandai S. B. Srinivasan H. (2019). Anthracnose disease diagnosis by image processing, support vector machine and correlation with pigments. J. Plant Pathol. 101, 749–751. doi: 10.1007/s42161-019-00268-9

[ref68] Kim Y. K. Wang Y. Liu Z. M. Kolattukudy P. E. (2002). Identification of a hard surface contact-induced gene in *Colletotrichum gloeosporioides* conidia as a sterol glycosyl transferase, a novel fungal virulence factor. Plant J. 30, 177–187. doi: 10.1046/j.1365-313x.2002.01284.x, PMID: 12000454

[ref69] Kramer-Haimovich H. Servi E. Katan T. Rollins J. Okon Y. Prusky D. (2006). Effect of ammonia production by *Colletotrichum gloeosporioides* on pelB activation, pectate lyase secretion, and fruit pathogenicity. Appl. Environ. Microbiol. 72, 1034–1039. doi: 10.1128/AEM.72.2.1034-1039.2006, PMID: 16461646 PMC1392887

[ref70] Kumari C. Sharma M. Kumar V. Sharma R. Kumar V. Sharma P.. (2022). Genome editing technology for genetic amelioration of fruits and vegetables for alleviating post-harvest loss. Bioengineering 9:176. doi: 10.3390/bioengineering9040176, PMID: 35447736 PMC9028506

[ref71] Kumari P. Singh R. Punia R. (2017). Survival of *Colletotrichum gloeosporioides* causing anthracnose of mango at different depths and durations in soil. J. Pharmacogn. Phytochem. 6, 2194–2198.

[ref72] Kunta M. Park J. W. Vedasharan P. da Graça J. V. Terry M. D. (2018). First report of *Colletotrichum queenslandicum* on persian lime causing leaf anthracnose in the United States. Plant Dis. 102:677. doi: 10.1094/PDIS-09-17-1382-PDN

[ref73] Lai A. A. Simon S. (2013). Post harvest management of anthracnose rot of mango (*Mangifera indica* l.). Ann. Plant Prot. Sci. 21, 121–124.

[ref74] Leadbeater A. J. (2014). Plant health management: fungicides and antibiotics, encyclopedia of agriculture and food systems. Biology. doi: 10.1016/B978-0-444-52512-3.00179-0

[ref75] Li Q. Bu J. Shu J. Yu Z. Tang L. Huang S.. (2019). *Colletotrichum* species associated with mango in southern China. Sci. Rep. 9:18891. doi: 10.1038/s41598-019-54809-4, PMID: 31827115 PMC6906457

[ref76] Li Q. Shu J. Tang L. Huang S. Guo T. Mo J.. (2020). First report of mango leaf anthracnose caused by *Colletotrichum asianum* in Vietnam. Plant Dis. 104:1558. doi: 10.1094/PDIS-09-19-1830-PDN

[ref77] Liang Y. S. Fu J. Y. Chao S. H. Tzean Y. Hsiao C. Y. Yang Y. Y.. (2022). Postharvest application of *Bacillus amyloliquefaciens* PMB04 fermentation broth reduces anthracnose occurrence in mango fruit. Agriculture 12:1646. doi: 10.3390/agriculture12101646

[ref78] Lima N. B. De Batista M. V. A. De Morais M. A. Barbosa M. A. Michereff S. J. Hyde K. D.. (2013a). Five *Colletotrichum* species are responsible for mango anthracnose in northeastern Brazil. Fungal Divers. 61, 75–88. doi: 10.1007/s13225-013-0237-6

[ref79] Lima N. B. Lima W. G. Tovar-Pedraza J. M. Michereff S. J. Câmara M. P. (2015). Comparative epidemiology of *Colletotrichum* species from mango in northeastern Brazil. Eur. J. Plant Pathol. 141, 679–688. doi: 10.1007/s10658-014-0570-y

[ref80] Lima N. B. Marques M. W. Michereff S. J. Morais M. A. Jr. Barbosa M. A. G. Camara M. P. S. (2013b). First report of mango anthracnose caused by *Colletotrichum karstii* in Brazil. Plant Dis. 97:1248. doi: 10.1094/PDIS-01-13-0002-PDN, PMID: 30722427

[ref81] Lin C. H. Long X. P. Li Z. P. Zhang Y. He J. J. Liu W. B.. (2020). First report of anthracnose of *Clausena lansium* caused by *Colletotrichum scovillei* in China. Plant Dis. 104:1557. doi: 10.1094/PDIS-08-19-1765-PDN

[ref82] Lin T. Xu X. F. Dai D. J. Shi H. J. Wang H. D. Zhang C. Q. (2016). Differentiation in development of benzimidazole resistance in *Colletotrichum gloeosporioides* complex populations from strawberry and grape hosts. Australas. Plant Pathol. 45, 241–249. doi: 10.1007/s13313-016-0413-8

[ref83] Lisboa D. O. Silva M. A. Pinho D. B. Pereira O. L. Furtado G. Q. (2018). Diversity of pathogenic and endophytic *Colletotrichum* isolates from *Licania tomentosa* in Brazil. For. Pathol. 48:e12448. doi: 10.1111/efp.12448

[ref84] Liu Y. An F. Zhang Y. Fu C. Su Y. (2021). First report of anthracnose on Jerusalem cherry caused by *Colletotrichum liaoningense* in Shandong, China. Plant Dis. 105:2248. doi: 10.1094/PDIS-01-21-0124-PDN

[ref85] Liu F. Damm U. Cai L. Crous P. W. (2013). Species of the *Colletotrichum gloeosporioides* complex associated with anthracnose diseases of Proteaceae. Fungal Divers. 61, 89–105. doi: 10.1007/s13225-013-0249-2

[ref86] Liu H. N. Liu J. A. Zhou G. Y. (2020). First report of *Colletotrichum alienum* causing anthracnose on *Aquilaria sinensis* in China. Plant Dis. 104:283. doi: 10.1094/PDIS-01-19-0155-PDN

[ref87] Liu L. P. Shu J. Zhang L. Hu R. Chen C. Q. Yang L. N.. (2017). First report of post-harvest anthracnose on mango (*Mangifera indica*) caused by *Colletotrichum siamense* in China. Plant Dis. 101:833. doi: 10.1094/PDIS-08-16-1130-PDN

[ref88] Lombard V. Golaconda Ramulu H. Drula E. Coutinho P. M. Henrissat B. (2014). The carbohydrate-active enzymes database (CAZy) in 2013. Nucleic Acids Res. 42, D490–D495. doi: 10.1093/nar/gkt1178, PMID: 24270786 PMC3965031

[ref89] Lopes L. H. R. Boiteux L. S. Rossato M. Aguiar F. M. Fonseca M. E. Oliveira V. R.. (2021). Diversity of *Colletotrichum* species causing onion anthracnose in Brazil. Eur. J. Plant Pathol. 159, 339–357. doi: 10.1007/s10658-020-02166-8

[ref90] Mabbett T. (2014). Disease management in mango. Int. Pest Control 56:104.

[ref91] Maharaj A. Rampersad S. N. (2012). Genetic differentiation of *Colletotrichum gloeosporioides* and *C. truncatum* associated with anthracnose disease of papaya (*Carica papaya* L.) and bell pepper (*Capsium annuum* L.) based on ITS PCR-RFLP fingerprinting. Mol. Biotechnol. 50, 237–249. doi: 10.1007/s12033-011-9434-2 21720933

[ref92] Malik M. T. Ammar M. Ranan M. Rehman A. Bally I. S. (2014). Chemical and cultural management of die back disease of mango in Pakistan. In XXIX International Horticultural Congress on Horticulture: Sustaining Lives, Livelihoods and Landscapes (IHC2014): IV 1111 (pp. 363–368).

[ref93] Manzano León A. M. Serra Hernández W. García Pérez L. Crespo K. Guarnaccia V. (2018). First report of leaf anthracnose caused by *Colletotrichum grossum* on mango (*Mangifera indica*) in Cuba. J. Plant Pathol. 100:329. doi: 10.1007/s42161-018-0040-z

[ref94] Marcianò D. Mizzotti C. Maddalena G. Toffolatti S. L. (2021). The dark side of Fungi: how they cause diseases in plants. Front. Young Minds 9:315. doi: 10.3389/frym.2021.560315

[ref95] Marikar F. M. M. T. Sivakumar D. Wijerathnam R. W. (2008). Biological control of rambutan post-harvest anthracnose (*Colleotrichum gloeosporioides*) by combined treatment of *Trichoderma harzianum*-TrH40 culture filtrates and calcium salts. Food Biotechnol. 22, 326–337. doi: 10.1080/08905430802458412

[ref96] Maske J. M. Masih S. Verma O. P. (2022). A review on morphological and molecular characterization of *Colletotrichum* species associated with mango anthracnose in Konkan region of Maharashtra state. J. Pharm. Innov. 11, 1577–1581.

[ref97] Maymon M. Zveibil A. Pivonia S. Minz D. Freeman S. (2006). Identification and characterization of benomyl-resistant and-sensitive populations of *Colletotrichum gloeosporioides* from statice (*Limonium* spp.). Phytopathology 96, 542–548. doi: 10.1094/PHYTO-96-0542, PMID: 18944315

[ref98] McCulloch M. J. Gauthier N. W. Vaillancourt L. J. (2020). First report of bitter rot of apple caused by a *Colletotrichum* sp. in the *C. kahawae* clade in Kentucky. Plant Dis. 104:289. doi: 10.1094/PDIS-06-19-1247-PDN

[ref99] Mirshekari A. Ding P. Kadir J. Ghazali H. M. (2012). Effect of hot water dip treatment on postharvest anthracnose of banana var Berangan. Afr. J. Agric. Res. 7, 6–10. doi: 10.5897/AJAR11.056

[ref100] Miyara I. Shnaiderman C. Meng X. Vargas W. A. Diaz-Minguez J. M. Sherman A.. (2012). Role of nitrogen-metabolism genes expressed during pathogenicity of the alkalinizing *Colletotrichum gloeosporioides* and their differential expression in acidifying pathogens. Mol. Plant-Microbe Interact. 25, 1251–1263. doi: 10.1094/MPMI-01-12-0017-R, PMID: 22571816

[ref101] Mo J. Zhao G. Li Q. Solangi G. S. Tang L. Guo T.. (2018). Identification and characterization of *Colletotrichum* species associated with mango anthracnose in Guangxi, China. Plant Dis. 102, 1283–1289. doi: 10.1094/PDIS-09-17-1516-RE 30673569

[ref102] Moraes S. R. G. Tanaka F. A. O. Massola Júnior N. S. (2013). Histopathology of *Colletotrichum gloeosporioides* on guava fruits (*Psidium guajava* L.). Rev. Bras. Frutic. 35, 657–664. doi: 10.1590/S0100-29452013000200039

[ref103] Münch S. Lingner U. Floss D. S. Ludwig N. Sauer N. Deising H. B. (2008). The hemibiotrophic lifestyle of *Colletotrichum* species. J. Plant Physiol. 165, 41–51. doi: 10.1016/j.jplph.2007.06.008, PMID: 17765357

[ref104] Munir M. Amsden B. Dixon E. Vaillancourt L. Gauthier N. W. (2016). Characterization of *Colletotrichum* species causing bitter rot of apple in Kentucky orchards. Plant Dis. 100, 2194–2203. doi: 10.1094/PDIS-10-15-1144-RE, PMID: 30682908

[ref105] Nelson S. C. (2008). Mango anthracnose (*Colletotrichum gloeosporiodes*). Plant Dis. 48, 1–9.

[ref106] Nesher I. Minz A. Kokkelink L. Tudzynski P. Sharon A. (2011). Regulation of pathogenic spore germination by CgRac1 in the fungal plant pathogen *Colletotrichum gloeosporioides*. Eukaryot. Cell 10, 1122–1130. doi: 10.1128/EC.00321-10, PMID: 21460190 PMC3165446

[ref107] Ntsoane M. L. Zude-Sasse M. Mahajan P. Sivakumar D. (2019). Quality assesment and postharvest technology of mango: A review of its current status and future perspectives. Sci. Hortic. 249, 77–85. doi: 10.1016/j.scienta.2019.01.033

[ref108] Oh B. J. Ko M. K. Kim Y. S. Kim K. S. Kostenyuk I. Kee H. K. (1999). A cytochrome P450 gene is differentially expressed in compatible and incompatible interactions between pepper (*Capsicum annuum*) and the anthracnose fungus, *Colletotrichum gloeosporioides*. Mol. Plant-Microbe Interact. 12, 1044–1052. doi: 10.1094/MPMI.1999.12.12.1044, PMID: 10624013

[ref109] Onyeani C. A. Amusa N. A. (2015). Incidence and severity of anthracnose in mango fruits and its control with plant extracts in south West Nigeria. Int. J. Agric. Res. 10, 33–43. doi: 10.3923/IJAR.2015.33.43

[ref110] Pardo-De la Hoz C. J. Calderón C. Rincón A. M. Cárdenas M. Danies G. López-Kleine L.. (2016). Species from the *Colletotrichum acutatum, Colletotrichum boninense* and *Colletotrichum gloeosporioides* species complexes associated with tree tomato and mango crops in Colombia. Plant Pathol. 65, 227–237. doi: 10.1111/ppa.12410

[ref111] Patel B. Tandel Y. N. Patel A. H. Patel B. L. (2016). Chilling injury in tropical and subtropical fruits: A cold storage problem and its remedies: A review. Int. J. Environ. Sci. Technol. 5, 1882–1887.

[ref112] Patil R. Y. Gulvani S. Waghmare V. B. Mujawar I. K. (2022). Image based anthracnose and red-rust leaf disease detection using deep learning. Telkomnika 20, 1256–1263. doi: 10.12928/telkomnika.v20i6.24262

[ref113] Patrice N. D. Alain H. Bertrand M. S. Norbert K. T. Nourou K. N. Brice T. T.. (2020). First report of red rust disease caused by *Cephaleuros virescens* on mango (*Mangifera indica*) tree in Cameroon. Int. J. Phytopathol. 9, 187–193. doi: 10.33687/phytopath.009.03.3432

[ref114] Paudel A. Poudel P. Yogi M. (2022). Insights on the mango anthracnose and its management. J Plant Pathol. Res. 4, 81–90. doi: 10.36959/394/629

[ref115] Paulitz T. C. (1995). First report of *Colletotrichum gloeosporioides* on lupine in Canada. Plant Dis. 79:319. doi: 10.1094/PD-79-0319D

[ref116] Peres N. A. Kuramae E. E. Dias M. S. De Souza N. L. (2002). Identification and characterization of *Colletotrichum* spp. affecting fruit after harvest in Brazil. J. Phytopathol. 150, 128–134. doi: 10.1046/j.1439-0434.2002.00732.x

[ref117] Pérez-Mora J. L. Mora-Romero G. A. Beltrán-Peña H. García-León E. Lima N. B. Camacho-Tapia M.. (2021). First report of *Colletotrichum siamense* and *C. gloeosporioides* causing anthracnose of Citrus spp. in Mexico. Plant Dis. 105:496. doi: 10.1094/PDIS-08-20-1743-PDN

[ref118] Prabu M. Chelliah B. J. (2022). Mango leaf disease identification and classification using a CNN architecture optimized by crossover-based levy flight distribution algorithm. Neural Comput. Appl. 34, 7311–7324. doi: 10.1007/s00521-021-06726-9

[ref119] Prakash O. Srivastava K. C. (1987). Mango diseases and their management. A world review. Today and Tomorrow's Printers and Publishers: New Delhi.

[ref120] Qin L. P. Zhang Y. Su Q. Chen Y. L. Nong Q. Xie L.. (2019). First report of anthracnose of *Mangifera indica* caused by *Colletotrichum scovillei* in China. Plant Dis. 103:1043. doi: 10.1094/PDIS-11-18-1980-PDN

[ref121] Reyes-Perez J. J. Hernandez-Montiel L. G. Vero S. Noa-Carrazana J. C. Quiñones-Aguilar E. E. Rincón-Enríquez G. (2019). Postharvest biocontrol of *Colletotrichum gloeosporioides* on mango using the marine bacterium *Stenotrophomonas rhizophila* and its possible mechanisms of action. J. Food Sci. Technol. 56, 4992–4999. doi: 10.1007/s13197-019-03971-8, PMID: 31741523 PMC6828899

[ref122] Rodriguez-Moreno L. Ebert M. K. Bolton M. D. Thomma B. P. (2018). Tools of the crook-infection strategies of fungal plant pathogens. Plant J. 93, 664–674. doi: 10.1111/tpj.13810, PMID: 29277938

[ref123] Rosman N. F. Asli N. A. Abdullah S. Rusop M. (2019). “Some common disease in mango” in AIP conference proceedings, vol. 2151 (New York: AIP Publishing LLC), 020019.

[ref124] Saeed E. E. Sham A. AbuZarqa A. Al Shurafa K. A. Al Naqbi T. S. Iratni R.. (2017). Detection and management of mango dieback disease in the United Arab Emirates. Int. J. Mol. Sci. 18:2086. doi: 10.3390/ijms18102086, PMID: 29053600 PMC5666768

[ref125] Sanchez-Arizpe A. Galindo-Cepeda M. E. Arispe-Vazquez J. L. Genis-Velazquez R. Vazquez-Badillo M. E. Antonio-Bautista A. (2021). Natural resistance of two Mango'*mangifera indica*'l. commercial cultivars to anthracnose caused by'Colletotrichum gloeosporioides' Penz. Penz. and Sacc. Aust. J. Crop Sci. 15, 1198–1203. doi: 10.21475/ajcs.21.15.08.p3347

[ref126] Sardrood B. P. Goltapeh E. M. (2018). “Effect of agricultural chemicals and organic amendments on biological control Fungi” in Sustainable Agriculture Reviews. ed. Lichtfouse E., vol. 12 (Berlin: Springer), 217–359.

[ref127] Sardsud U. Sardsud V. Singkaew S. (2003). Postharvest loss assessment of mango cv. Nam Dok Mai. Warasan Witthayasat Kaset. Available at: https://agris.fao.org/agris-search/search.do?recordID=TH2005000687 (Accessed February 1, 2023).

[ref128] Sarkar A. K. (2016). Anthracnose diseases of some common medicinally important fruit plants. J. Med. Plant Res. 4, 233–236.

[ref129] Schena L. Mosca S. Cacciola S. O. Faedda R. Sanzani S. M. Agosteo G. E.. (2014). Species of the *Colletotrichum gloeosporioides* and *C. boninense* complexes associated with olive anthracnose. Plant Pathol. 63, 437–446. doi: 10.1111/ppa.12110

[ref130] Sefu G. Satheesh N. Berecha G. (2015). Effect of essential oils treatment on anthracnose (*Colletotrichum gloeosporioides*) disease development, quality and shelf life of mango fruits (*Mangifera indica* L). Am. Eurasian J. Agric. Environ. Sci. 15, 2160–2169. doi: 10.5829/idosi.aejaes.2015.15.11.96140

[ref131] Serrato-Diaz L. M. Rivera-Vargas L. I. French-Monar R. D. (2014). First report of *Neofusicoccum mangiferae* causing rachis necrosis and inflorescence blight of mango (*Mangifera indica*) in Puerto Rico. Plant Dis. 98:570. doi: 10.1094/PDIS-08-13-0878-PDN, PMID: 30708705

[ref132] Sharma M. Kulshrestha S. (2015). *Colletotrichum gloeosporioides*: an anthracnose causing pathogen of fruits and vegetables. Biosci. Biotechnol. Res. Asia 12, 1233–1246. doi: 10.13005/bbra/1776

[ref133] Sharma G. Kumar N. Weir B. S. Hyde K. D. Shenoy B. D. (2013). The ApMat marker can resolve *Colletotrichum* species: a case study with *Mangifera indica*. Fungal Divers. 61, 117–138. doi: 10.1007/s13225-013-0247-4

[ref134] Sharma G. Maymon M. Freeman S. (2017). Epidemiology, pathology and identification of *Colletotrichum* including a novel species associated with avocado (*Persea americana*) anthracnose in Israel. Sci. Rep. 7:15839. doi: 10.1038/s41598-017-15946-w, PMID: 29158592 PMC5696532

[ref135] Sharma A. Verma K. S. (2007). In vitro cross pathogenicity and management of *Colletotrichum gloeosporioides* causing anthracnose of mango. Ann. Plant Prot. Sci 15, 186–188.

[ref136] Shi N. Ruan H. Jie Y. Chen F. Du Y. (2020). Sensitivity and efficacy of fungicides against wet bubble disease of Agaricus bisporus caused by *Mycogone perniciosa*. Eur. J. Plant Pathol. 157, 873–885. doi: 10.1007/s10658-020-02047-0

[ref137] Shih J. Wei Y. Goodwin P. H. (2000). A comparison of the pectate lyase genes, pel-1 and pel-2, of *Colletotrichum gloeosporioides* f. sp. malvae and the relationship between their expression in culture and during necrotrophic infection. Gene 243, 139–150. doi: 10.1016/s0378-1119(99)00546-6, PMID: 10675622

[ref138] Shivas R. G. Tan Y. P. Edwards J. Dinh Q. Maxwell A. Andjic V.. (2016). *Colletotrichum* species in Australia. Australas. Plant Pathol. 45, 447–464. doi: 10.1007/s13313-016-0443-2

[ref139] Silué Y. Nindjin C. Cissé M. Kouamé K. A. Mbéguié-A-Mbéguié D. Lopez-Lauri F.. (2022). Hexanal application reduces postharvest losses of mango (*Mangifera indica* L. variety" Kent") over cold storage whilst maintaining fruit quality. Postharvest Biol. Technol. 189:111930. doi: 10.1016/j.postharvbio.2022.111930

[ref140] Siriptrawan U. (2021). Early detection of anthracnose on mango fruit using hyperspectral imaging. Rep. Grant. Res. Asahi Glass Foundation 90:81. doi: 10.50867/afreport.2021_081

[ref141] Sosnowski M. R. Fletcher J. D. Daly A. M. Rodoni B. C. Viljanen-Rollinson S. L. H. (2009). Techniques for the treatment, removal and disposal of host material during programmes for plant pathogen eradication. Plant Pathol. 58, 621–635. doi: 10.1111/j.1365-3059.2009.02042.x

[ref142] Stephenson S. A. Hatfield J. Rusu A. G. Maclean D. J. Manners J. M. (2000). CgDN3: an essential pathogenicity gene of *Colletotrichum gloeosporioides* necessary to avert a hypersensitive-like response in the host *Stylosanthes guianensis*. Mol. Plant-Microbe Interact. 13, 929–941. doi: 10.1094/MPMI.2000.13.9.929 10975650

[ref143] Su Y. Y. Noireung P. Liu F. Hyde K. D. Moslem M. A. Bahkali A. H.. (2011). Epitypification of *Colletotrichum musae*, the causative agent of banana anthracnose. Mycoscience 52, 376–382. doi: 10.1007/s10267-011-0120-9

[ref144] Supriya A. Kumar A. Kudachikar V. B. (2020). A comparison investigation on antioxidant activities, constitutive antifungal phenolic lipids and phenolics contents of anthracnose resistant and susceptible mango fruit cultivars. Int. J. Fruit Sci. 20, 692–704. doi: 10.1080/15538362.2019.1668332

[ref145] Talhinhas P. Baroncelli R. (2021). *Colletotrichum* species and complexes: geographic distribution, host range and conservation status. Fungal Divers. 110, 109–198. doi: 10.1007/s13225-021-00491-9

[ref146] Talhinhas P. Loureiro A. Oliveira H. (2018). Olive anthracnose: a yield-and oil quality-degrading disease caused by several species of *Colletotrichum* that differ in virulence, host preference and geographical distribution. Mol. Plant Pathol. 19, 1797–1807. doi: 10.1111/mpp.12676, PMID: 29517840 PMC6638118

[ref147] Tashiro N. Manabe K. Ide Y. (2012). Emergence and frequency of highly benzimidazole-resistant Colletotrichum gloeosporioides, pathogen of Japanese pear anthracnose, after discontinued use of benzimidazole. J. Gen. Plant Pathol. 78, 221–226. doi: 10.1007/s10327-012-0375-9

[ref148] Theerthagiri A. Govindasamy S. Thiruvengadam R. Ramasamy S. (2016). Immunoassay-based techniques for early and specific detection of latent postharvest anthracnose in mango. J. Phytopathol. 164, 318–329. doi: 10.1111/jph.12459

[ref149] Thind T. S. Hollomon D. W. (2018). Thiocarbamate fungicides: reliable tools in resistance management and future outlook. Pest Manag. Sci. 74, 1547–1551. doi: 10.1002/ps.4844, PMID: 29286551

[ref150] Torres-Calzada C. Tapia-Tussell R. Higuera-Ciapara I. Perez-Brito D. (2013). Morphological, pathological and genetic diversity of *Colletotrichum* species responsible for anthracnose in papaya (*Carica papaya* L). Eur. J. Plant Pathol. 135, 67–79. doi: 10.1007/s10658-012-0065-7

[ref151] Tovar-Pedraza J. M. Mora-Aguilera J. A. Nava-Diaz C. Lima N. B. Michereff S. J. Sandoval-Islas J. S.. (2020). Distribution and pathogenicity of *Colletotrichum* species associated with mango anthracnose in Mexico. Plant Dis. 104, 137–146. doi: 10.1094/PDIS-01-19-0178-RE, PMID: 31730415

[ref152] Tucho A. Lemessa F. Berecha G. (2014). Distribution and occurrence of mango anthracnose (*Colletotrichum gloesporioides* penz and sacc) in humid agro-ecology of Southwest Ethiopia. Plant Pathol. J. 13, 268–277. doi: 10.3923/ppj.2014.268.277

[ref153] Uddin M. Shefat S. Afroz M. Moon N. (2018). Management of anthracnose disease of mango caused by *Colletotrichum gloeosporioides*: A review. Acta Sci. Agric. 2, 169–177. doi: 10.35248/2157-7471.22.13.635

[ref154] Ullagaddi S. B. Raju S. V. (2017). “Disease recognition in mango crop using modified rotational kernel transform features” in 2017 4th international conference on advanced computing and communication systems (ICACCS) (New York: IEEE), 1–8.

[ref155] Veling P. S. Kalelkar R. S. Ajgaonkar L. V. Mestry N. V. Gawade N. N. (2019). Mango disease detection by using image processing. IJRASET 7, 3717–3726. doi: 10.22214/ijraset.2019.4624

[ref156] Veloso J. S. Câmara M. P. Lima W. G. Michereff S. J. Doyle V. P. (2018). Why species delimitation matters for fungal ecology: *Colletotrichum* diversity on wild and cultivated cashew in Brazil. Fungal boil. 122, 677–691. doi: 10.1016/j.funbio.2018.03.005, PMID: 29880203

[ref157] Vilcarromero-Ramos R. L. Díaz-Valderrama J. R. Caetano A. C. Huamán-Pilco J. Mansilla-Córdova P. J. (2022). First report of anthracnose caused by *Colletotrichum asianum* on mango (*Mangifera indica*) in Peru. Plant Dis. 107:2245. doi: 10.1094/PDIS-10-22-2357-PDN, PMID: 36471463

[ref158] Vitale A. Alfenas A. C. de Siqueira D. L. Magistà D. Perrone G. Polizzi G. (2020). Cultivar resistance against *Colletotrichum asianum* in the world collection of mango germplasm in southeastern Brazil. Plan. Theory 9:182. doi: 10.1094/PDIS.2000.84.6.600 PMC7076395 32024312

[ref159] Wang Y. C. Hao X. Y. Wang L. Xiao B. Wang X. C. Yang Y. J. (2016). Diverse *Colletotrichum* species cause anthracnose of tea plants (*Camellia sinensis* (L.) O. Kuntze) in China. Sci. Rep. 6:35287. doi: 10.1038/srep35287 27782129 PMC5080629

[ref160] Wei Y. Shih J. Li J. Goodwin P. H. (2002). Two pectin lyase genes, pnl-1 and pnl-2, from *Colletotrichum gloeosporioides* f. sp. malvae differ in a cellulose-binding domain and in their expression during infection of *Malva pusilla*. Microbiol. 148, 2149–2157. doi: 10.1099/00221287-148-7-2149 12101302

[ref161] Weir B. S. Johnston P. R. Damm U. (2012). The *Colletotrichum gloeosporioides* species complex. Stud. Mycol. 73, 115–180. doi: 10.3114/sim0011, PMID: 23136459 PMC3458417

[ref162] Wu Y. Cheng J. H. Sun D. W. (2022). Subcellular damages of *Colletotrichum asianum* and inhibition of mango anthracnose by dielectric barrier discharge plasma. Food Chem. 381:132197. doi: 10.1016/j.foodchem.2022.132197 35121319

[ref163] Wu J. Ji Z. Wang N. Chi F. Xu C. Zhou Z.. (2016). Identification of conidiogenesis-associated genes in *Colletotrichum gloeosporioides* by *Agrobacterium tumefaciens*-mediated transformation. Curr. Microbiol. 73, 802–810. doi: 10.1007/s00284-016-1131-8, PMID: 27582094

[ref164] Yokosawa S. Eguchi N. Kondo K. I. Sato T. (2017). Phylogenetic relationship and fungicide sensitivity of members of the *Colletotrichum gloeosporioides* species complex from apple. J. Gen. Plant Pathol. 83, 291–298. doi: 10.1007/s10327-017-0732-9

[ref165] Yong H. Y. Bakar F. D. A. Illias R. M. Mahadi N. M. Murad A. A. (2013). Cgl-SLT2 is required for appressorium formation, sporulation and pathogenicity in *Colletotrichum gloeosporioide*. Braz. J. Microbiol. 44, 1241–1250. doi: 10.1590/S1517-83822013000400031, PMID: 24688518 PMC3958194

[ref166] Zakaria L. Juhari N. Z. Vijaya S. I. Anuar I. S. M. (2015). Molecular characterization of *Colletotrichum* isolates associated with anthracnose of mango fruit. Sains Malays. 44, 651–656. doi: 10.17576/jsm-2015-4405-02

[ref167] Zhafarina S. Wibowo A. Widiastuti A. (2021). Multi-genetic analysis of *Colletotrichum* spp. associated with postharvest disease of fruits anthracnose in special region of Yogyakarta, Indonesia. Pak. J. Biol. Sci. PJBS 24, 53–65. doi: 10.3923/pjbs.2021.53.65, PMID: 33683031

[ref168] Zhang H. Wei Y. Shi H. (2020). First report of anthracnose caused by *Colletotrichum kahawae* subsp. ciggaro on Areca in China. Plant Dis. 104:1871. doi: 10.1094/PDIS-12-19-2628-PDN

[ref169] Zhou Y. Huang J. S. Yang L. Y. Wang G. F. Li J. Q. (2017). First report of banana anthracnose caused by *Colletotrichum scovillei* in China. Plant Dis. 101:381. doi: 10.1094/PDIS-08-16-1135-PDN

[ref170] Zhou Z. Wu J. Wang M. Zhang J. (2017). ABC protein CgABCF2 is required for asexual and sexual development, appressorial formation and plant infection in *Colletotrichum gloeosporioides*. Microb. Pathog. 110, 85–92. doi: 10.1016/j.micpath.2017.06.028 28645773

[ref171] Zin N. A. Badaluddin N. A. (2020). Biological functions of *Trichoderma* spp. for agriculture applications. Ann. Agric. Sci. 65, 168–178. doi: 10.1016/j.aoas.2020.09.003

