# Supplementary material for: Mango anthracnose disease: the current situation and direction for future research
Source: Front Microbiol. 2023 Aug 24;14:1168203. doi: 10.3389/fmicb.2023.1168203 (PMC10484599; doi:10.3389/fmicb.2023.1168203)
Supplement: Supplementary file 1 [file Table_1.DOCX]

Table S1: *Colletotrichum gloeosporiodes* phenotypes derived from analysis of evolved DNA sequences within the ITS region.

| **Species name** | **Culture number** | **Origin of culture** |  | **Host** | **Origin of phenotype** | **GenBank Accession number(s)** | **Reference** |
| --- | --- | --- | --- | --- | --- | --- | --- |
| *C. aeschynomenes* | ^1^ICMP 17673 | ^3^HT |  | *Aeschynomene virginica*  (Sensitive-joint vetch) | USA | **ITS**: JX010176; ***tub2***: JX010392; ***act***: JX009483; ***chs 1***: JX009799; ***gapdh***: JX009930; ***cal***: JX009721; ***gs***: JX010081; ***sod2***: JX010314 | Weir *et al*., 2012 |
| *C. fruticola* | ICMP 17921 | HT |  | *Ficus edulis* (Figs) | Germany | **ITS**: FJ972603, JX010165; ***tub2***: FJ907441, JX010405; ***act***: FJ907426; ***chs 1***: JX009866; ***gapdh***: FJ972578, JX010033; ***cal***: FJ917508; ***gs***: FJ972593, JX010095; ***sod2***: JX010327 | Prihastuti *et al*. (2009), Weir *et al*. (2012) |
| *C. nupharicola* | ICMP 18187 | HT |  | *Nuphar lutea subsp. Polysepala*  (Pond-lily) | USA | **ITS**: JX010187; ***tub2***: JX010398; ***act***: JX009437; ***chs 1***: JX009835; ***gapdh***: JX009972; ***cal***: JX009663; ***gs***: JX010088; ***sod2***: JX010320 | Weir *et al*., 2012 |
| *C. alienum* | ICMP 12071 | HT |  | *Malus domestica*  (Apple) | New Zealand | **ITS**: JX010251; ***tub2***: JX010411; ***act***: JX009572; ***chs 1***: JX009882; ***gapdh***: JX010028; ***cal***: JX009654; ***gs***: JX010101; ***sod2***: JX010333 | Weir *et al*., 2012 |
| *C. queenslandicum* | ICMP 1778 | ^4^ET |  | *Carica papaya*  (Pawpaw/papaya) | Australia | **ITS**: JX010276; ***tub2***: JX010414; ***act***: JX009447; ***chs 1***: JX009899; ***gapdh***: JX009934; ***cal***: JX009691; ***gs***: JX010104; ***sod2***: JX010336 | Weir *et al*. (2012) |
| *C. salsolae* | ICMP 19051 | HT |  | *Salsola tragus*  (Prickly Russian thistle) | Hungary | **ITS**: JX010242; ***tub2***: JX010403; ***act***: JX009562; ***chs 1***: JX009863; ***gapdh***: JX009916; ***cal***: JX009696; ***gs***: JX010093; ***sod2***: JX010325 | Weir *et al*. (2012) |
| *C. asianum* | ICMP 18580 | HT |  | *Coffea arabica* (Coffee) | Thailand | **ITS**: FJ972612; ***tub2***: JX010406; ***act***: JX009584; ***chs 1***: JX009867; ***gapdh***: JX010053; ***cal***: FJ917506; ***gs***: JX010096; ***sod2***: JX010328 | Prihastuti *et al*. (2009), Weir *et al*. (2012) |
| *C. siamense* | ^2^MTCC 11590 | HT |  | *Mangifera indica* | India | **ITS**: JQ894658; ***tub2***: JQ894590; ***act***: JQ894534; ***chs 1***: JQ894605; ***gapdh***: JQ894620; ***cal***: KC790780 | Sharma *et al*., (2013) |
| *C. aenigma* | ICMP 18608 | HT |  | *Persea americana* (Avocado) | Israel | **ITS**: JX010244; ***tub2***: JX010389; ***act***: JX009443; ***chs 1***: JX009774; ***gapdh***: JX010044; ***cal***: JX009683; ***gs***: JX010078; ***sod2***: JX010311 | Weir *et al*., 2012 |
| *C. tropicale* | ICMP 18653 | HT |  | *Theobroma cacao* (Cocoa) | Panama | **ITS**: GU994331, JX010264; ***tub2***: GU994454, JX010407; ***act***: JX009489; ***chs 1***: JX009870; ***gapdh***: JX010007; ***cal***: JX009719; ***gs***: JX010097; ***sod2***: JX010329; ***Mat1/apn2***: GU994425; ***apn2***: GU994396; ***tef1***: GU994483 | Rojas *et al*. (2010), Weir *et al*. (2012) |
| *C. musae* | ICMP 19119 | ET |  | *Musa sp.* (Banana) | USA | **ITS**: HQ596292, JX010146; ***tub2***: HQ596280; ***act***: HQ596284, JX009433; ***chs 1***: JX009896; ***gapdh***: HQ596299, JX010050; ***cal***: JX009742; ***gs***: HQ596288, JX010103; ***sod2***: JX010335 | Su *et al*. (2011), Weir *et al*. (2012) |
| *C. gloeosporioides* | ICMP 17821 | ET |  | *Citrus sinensis*  (Sweet orange) | Italy | **ITS**: EU371022, JQ005152, JX010152; ***tub2***: FJ907445, JQ005587, JX010445; ***act***: FJ907430, JQ005500, JX009531; ***chs 1***: JQ005326, JX009818; ***gapdh***: FJ972582, JQ005239, JX010056; ***his3***: JQ005413; ***cal***: FJ917512, JQ005673, JX009731; ***gs***: FJ972589, JX010085; ***sod2***: JX010365 | Damm *et al*. (2012), Weir *et al*. (2012) |
| *C. alatae* | ICMP 17919 | HT |  | *Dioscorea alata* (Yam) | India | **ITS**: JX010190; ***tub2***: JX010383; ***act***: JX009471; ***chs 1***: JX009837; ***gapdh***: JX009990; ***cal***: JX009738; ***gs***: JX010065; ***sod2***: JX01030 | Weir *et al*., 2012 |
| *C. xanthorrhoeae* | ICMP 17903 | HT |  | *Xanthorrhoea preissii* (Grass tree) | Australia | **ITS**: GU048667, GU174551, JX010261; ***tub2***: JX010448; ***act***: JX009478; ***chs 1***: JX009823; ***gapdh***: GU174563, JX009927; ***cal***: JX009653; ***gs***: JX010138; ***sod2***: JX010369; TEF1: GU174575 | Hyde *et al*. (2009), Weir and Johnston (2010), Weir *et al*. (2012) |
| *C. clidemiae* | ICMP 18658 | HT |  | *Clidemia hirta* (Clidemia/ soapbush) | USA, Hawaii | **ITS**: JX010265; ***tub2***: JX010438; ***act***: JX009537; ***chs 1***: JX009877; ***gapdh***: JX009989; ***cal***: JX009645; ***gs***: JX010129; ***sod2***: JX010356 | Weir *et al*., 2012 |
| *C. kahawae* | ICMP 17816 | HT |  | *Coffea arabica* (Coffee) | Kenya | **ITS**: GU174550, JX010231; ***tub2***: JX010444; ***act***: JX009452; ***chs 1***: JX009813; ***gapdh***: GU174562, JX010012; ***cal***: JX009642; ***gs***: JX010130; ***sod2***: JX010130 | Weir *et al*., 2012 |
| *C. pisidii* | ICMP 19120 | ^5^AUT |  | *Psidium* sp. (Guava) | Italy | **ITS**: JX010219; ***tub2***: JX010443; ***act***: JX009515; ***chs 1***: JX009901; ***gapdh***: JX009967; ***cal***: JX009743; ***gs***: JX010133; ***sod2***: JX010366 | Weir *et al*., 2012 |
| *C. cordynilicola* | ICMP 18579 | HT |  | *Cordyline fruticose*  (Ti plant) | Thailand | **ITS**: HM470246, JX010226; ***tub2***: HM470249, JX010440; ***act***: HM470234; ***chs 1***: JX009864; ***gapdh***: HM470240, JX009975; ***cal***: HM470237; ***gs***: HM470243, JX010122; ***sod2***: JX010361 | Phoulivong *et al*. (2010), Weir *et al*. (2012) |
| *C. aotearoa* | ICMP 18537 | HT |  | *Coprosma* sp.  (Coprosma plants) | New Zealand | **ITS**: JX010205; ***tub2***: JX010420; ***act***: JX009564; ***chs 1***: JX009853; ***gapdh***: JX010005; ***cal***: JX009611; ***gs***: JX010113; ***sod2***: JX010345 | Weir *et al*., 2012 |
| *C. ti* | ICMP 4832 | HT |  | *Cordyline* sp.  (Cabbage palm) | New Zealand | **ITS**: JX010269; ***tub2***: JX010442; ***act***: JX009520; ***chs 1***: JX009898; ***gapdh***: JX009952; ***cal***: JX009649; ***gs***: JX010123; ***sod2***: JX010362 | Weir *et al*. (2012) |
| *C. theobromicola* | ^5^ICMP 18649 | ^6^NT |  | *Theobroma cacao* (Cocoa) | Panama | **ITS**: GU994360, JX010294; ***tub2***: GU994477, JX010447; ***act***: JX009444; ***chs 1***: JX009869; ***gapdh***: JX010006; ***cal***: JX009591; ***gs***: JX010139; ***sod2***: JX010372; ***Mat1/apn2***: GU994448; ***apn2***: GU994419; TEF1: GU994506 | Rojas *et al*. (2010), Weir *et al*. (2012) |
| *C. horii* | ICMP 10492 | NT |  | *Diospyros kaki* (Japanese Persimmon) | Japan | **ITS**: GQ329690; ***tub2***: JX010450; ***act***: JX009438; ***chs 1***: JX009752; ***gapdh***: GQ329681; ***cal***: JX009604; ***gs***: JX010137; ***sod2***: JX010370; ***tef1***: GQ329693 | Weir and Johnston (2010), Weir *et al*. (2012) |

^1^ICMP: International Collection of Microorganisms from Plants, Landcare Research, Auckland, New Zealand, ^2^MTCC – Microbial Type Culture Collection and Gene Bank, Chandirga, India – the sources of the cultures.

^3^HT – ex-holotype, ^4^ET – epitype , ^5^AUT – authentic culture, ^6^NT – ex-neotype; are the state or type of the original culture.

References

Damm, U., Cannon, P. F., Woudenberg, J. H. C., Johnston, P. R., Weir, B. S., Tan, Y. P., et al., (2012). The *Colletotrichum boninense* species complex. *Stud. Mycol.* 73(1), 1-36. dio: 10.3114/sim0002

Hyde, K. D., Cai, L., McKenzie, E. H. C., Yang, Y. L., Zhang, J. Z. and Prihastuti, H. (2009). *Colletotrichum*: a catalogue of confusion. *Fungal Divers.* 39(1), 1-17.

Phoulivong, S., Cai, L., Chen, H., McKenzie, E. H., Abdelsalam, K., Chukeatirote, E., et al. (2010). *Colletotrichum gloeosporioides* is not a common pathogen on tropical fruits. *Fungal Divers.* 44, 33-43. dio: 10.1007/s13225-010-0046-0

Prihastuti, H., Cai, L., Chen, H., McKenzie, E. H. C., and Hyde, K. D. (2009). Characterization of *Colletotrichum* species associated with coffee berries in northern Thailand. *Fungal Divers*. 39(1), 89-109.

Rojas, E. I., Rehner, S. A., Samuels, G. J., Van Bael, S. A., Herre, E. A., Cannon, P., et al. (2010). *Colletotrichum gloeosporioides* sl associated with *Theobroma cacao* and other plants in Panama: multilocus phylogenies distinguish host-associated pathogens from asymptomatic endophytes. *Mycologia*. *102*(6), 1318-1338. dio: 10.3852/09-244

Sharma, G., Kumar, N., Weir, B. S., Hyde, K. D., and Shenoy, B. D. (2013). The ApMat marker can resolve *Colletotrichum* species: a case study with *Mangifera indica*. *Fungal Divers*. 61, 117-138. dio: 10.1007/s13225-013-0247-4

Su, Y. Y., Noireung, P., Liu, F., Hyde, K. D., Moslem, M. A., Bahkali, A. H., et al. (2011). Epitypification of *Colletotrichum musae*, the causative agent of banana anthracnose. *Mycoscience*. 52(6), 376-382. dio: 10.1007/s10267-011-0120-9

Weir, B. S., and Johnston, P. R. (2010). Characterisation and neotypification of *Gloeosporium kaki* Hori as *Colletotrichum horii* nom. nov. *Mycotaxon*. 111(1), 209-219.

Weir, B. S., Johnston, P. R. and Damm, U. (2012). The *Colletotrichum gloeosporioides* species complex. *Stud. Mycol.* 73:115-180. [doi: 10.3114/sim0011](https://doi.org/10.3114/sim0011)
